# Single-cell landscape reveals active cell subtypes and their interaction in the tumor microenvironment of gastric cancer

**DOI:** 10.7150/thno.71833

**Published:** 2022-05-09

**Authors:** Yumin Li, Xueda Hu, Ruichai Lin, Guangyu Zhou, Lulu Zhao, Dongbing Zhao, Yawei Zhang, Wenbin Li, Yueming Zhang, Peiqin Ma, Hu Ren, Xinhui Liao, Penghui Niu, Tongbo Wang, Xiaojie Zhang, Wanqing Wang, Ranran Gao, Qibin Li, George Church, Jie He, Yingtai Chen

**Affiliations:** 1 Lanzhou University Second Hospital, Lanzhou 730030, China.; 2 BIOPIC, Beijing Advanced Innovation Center for Genomics, School of Life Sciences, Peking University, Beijing 100871, China.; 3 Clabee Genomics, Urban Garden Building, Bookstore Road, Luohu District, Shenzhen 518000, Guangdong, China.; 4 Department of Genetics, Harvard Medical School, Boston, Massachusetts 02115, USA.; 5 Department of Molecular and Cellular Biology, Harvard University, Cambridge, Massachusetts 02138, United States.; 6 National Cancer Center/National Clinical Research Center for Cancer/Cancer Hospital, Chinese Academy of Medical Sciences and Peking Union Medical College, Beijing 100021, China.; 7 Center on Translational Neuroscience, College of Life and Environmental Sciences, Minzu University of China, Beijing 100081, China.; 8 Wyss Institute for Biologically Inspired Engineering, Harvard University, Boston, Massachusetts 02115, USA.

**Keywords:** Gastric cancer, tumor microenvironment, single-cell RNA sequencing.

## Abstract

**Background:** Gastric cancer remains the third most common cause of cancer-related death worldwide. The development of novel therapeutic strategies for gastric cancer requires a deep understanding of the tumor cells and microenvironment of gastric cancer.

**Methods:** We performed the single-cell RNA sequencing (scRNA-seq) on nine untreated non-metastatic gastric cancer patients. The transcriptomic atlas and ligand-receptor-based intercellular communication networks of the single cells were characterized.

**Results:** Here, we profiled the transcriptomes of 47,304 cells from nine patients with gastric cancer. Tregs cells were significantly enriched in the gastric tumor tissues with increased expression of immune suppression related genes, which suggest a more immunosuppressive microenvironment. We also observed the absence of separate exhausted CD8+ T cell cluster, and the low expression level of exhaustion markers PDCD1, CTLA4, HAVCR2, LAG-3, and TIGIT in those specific cells. These may serve as molecular-level evidence for the limited benefit of immunotherapy among gastric cancer patients. In addition, we found ACKR1 specifically expressed in tumor endothelial cells, associated with poor prognosis in the cohort data and potentially provided a novel target of gastric cancer treatment. Furthermore, the tight interaction between endothelial cells and fibroblast implied the important roles of fibroblast in tumor angiogenesis and the maintenance of tumor vasculature.

**Conclusions:** In conclusion, this single-cell atlas provide understanding the cellular heterogeneity from molecular level in gastric cancer and will serve as a valuable resource for developing innovative early and companion diagnostics, as well as discovering novel targeted therapies for gastric cancer.

## Background

Gastric cancer is the third leading cause of cancer-related mortality and the sixth most common type of cancer globally 1. The existing traditional treatment of gastric cancer has reached the therapeutic plateau, and it is urgent to find new breakthroughs. Though proven in other cancers, anti PD-L1 antibody (avelumab) did not bring hope to gastric cancer patients in a recent phase III trial, failing to meet its primary end point of improving overall survival 2. Meanwhile, a PD-1 inhibitor (nivolumab) showed limited survival benefit for advanced gastric cancer 3. Additional targets that have been explored include vascular endothelial growth factor (VEGF) and angiopoietin-1/2. Studies in animals have shown that blocking these proteins may inhibit cancer cell proliferation 4-6. But the survival outcomes of related clinical trials, combined with chemotherapy drugs, were still inconclusive 7. Therefore, there is an unprecedented need to deepen our understanding of the tumor microenvironment (TME) in gastric cancer to identify novel targets for improving the clinical management of this disease.

TME comprises various cell types and extra-cellular components that are surrounding tumor cells and nourished by a vascular network. The cellular heterogeneity within TME is extremely complex and the recent advances in single-cell RNA sequencing (scRNA-seq) have enabled the analysis at a single-cell resolution among various malignant and non-transformed cell types, which can impact cancer progression and metastasis. Costa et al. 8 have identified four subsets of carcinoma-associated fibroblasts (CAF) in breast cancer, and one subset of them promotes an immunosuppressive microenvironment by recruiting CD4+CD25+ T cells. Fang et al. 9 presented a subset of tumor-associated macrophages (TAM), PLTP+C1QC+ TAMs, which may regulate the abundance of dysfunctional T cells through cytokine/chemokine signaling. Thus, a deeper understanding of stromal and immune cells could offer insights to develop novel therapies that exploit the therapeutic vulnerabilities of the TME and reprogram TME components to control gastric cancer progression.

Previous scRNA-seq studies on gastric cancer have reported the characteristics of gastric epithelial cells across different lesions and tumor heterogeneity, however, little is known about the association among immunosuppressive microenvironment, interactions between specific cell types, such as immune and stromal cells, and cancer progression 10-13. In this study, we performed droplet based scRNA-seq on tumor tissues and matched normal tissues from nine untreated non-metastatic gastric cancer patients, aiming to depict the cellular composition for gastric cancer, identify the changes of gene expression for different subsets of immune and stromal cells in TME, and construct the cellular interaction network in gastric cancer.

## Results

### Single-cell RNA sequencing identified seven major cell types in gastric cancer

**Figure 1:**
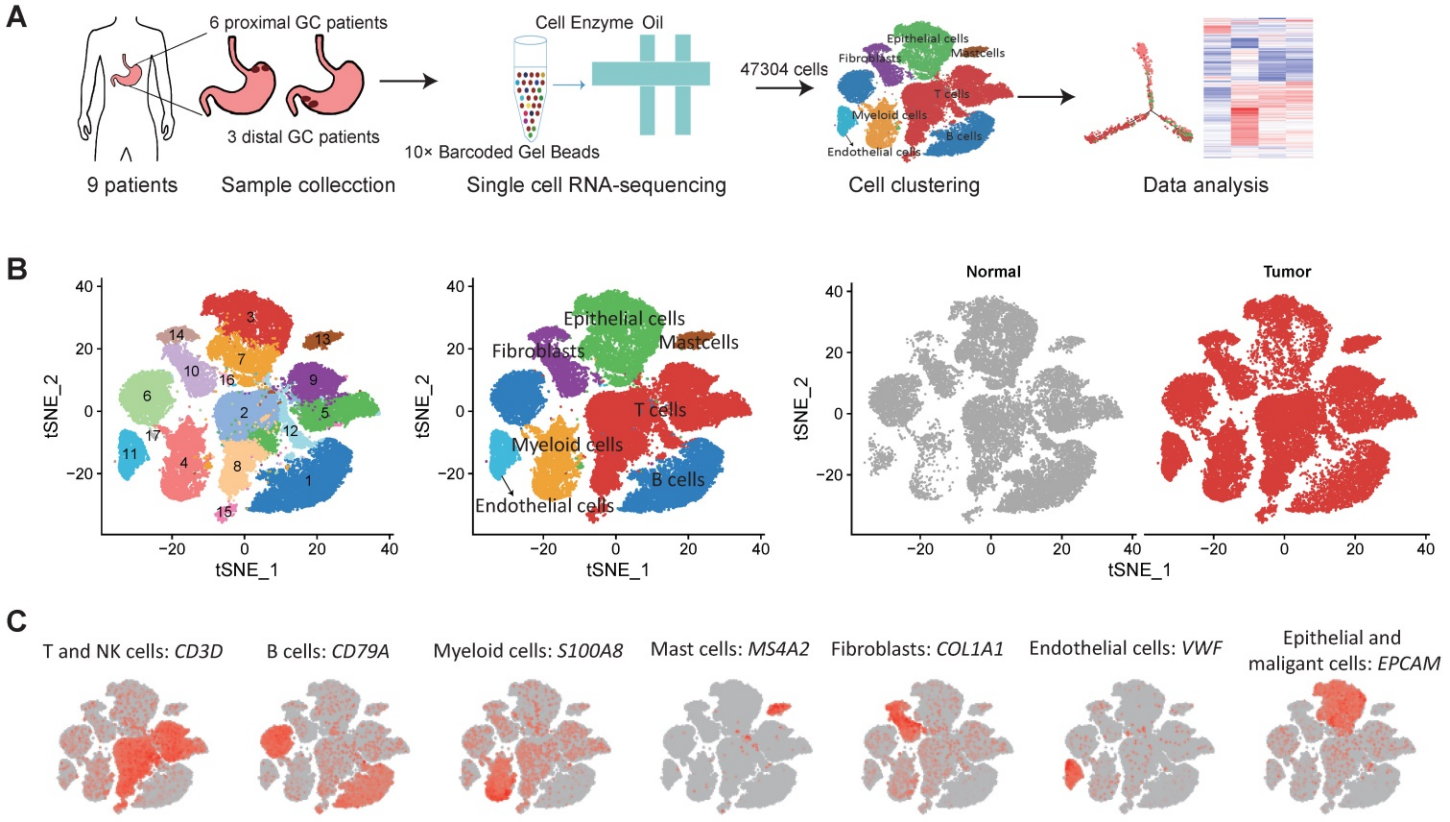
** Single cell RNA-seq of gastric tumor and adjacent non-malignant samples.** A, the design and workflow of this study. B, tSNE plots of cells from tumor and matched non-malignant samples of nine GC patients, colored by clusters (left panel), cell types (middle panel) and tissue origin (right panel). C, tSNE plots of known marker genes of each major cell type.

We collected fresh tumor samples and adjacent non-tumor samples from nine patients with untreated no metastatic gastric cancer who underwent gastrectomy with curative intent. Six of these patients had proximal gastric cancer (labeled P01-P06) and three had distal gastric cancer (labeled D01-D03) (Figure S1A-B). Detailed clinical and pathological information are provided in Table S1. Fresh samples collected during gastrectomy were rapidly digested into a single-cell suspension and analyzed using droplet-based single-cell transcriptome profiling (Figure 1A). A total of 47,304 cells with detectable expression of more than 200 genes were obtained after quality control (Methods), and 60.4% of these cells were derived from tumor samples. After the normalization of read counts and principal component analysis, we could obtain 17 cell clusters using graph-based clustering (Figure 1B, Methods). Based on the expression of canonical marker genes and top differently expressed gene of these clusters (Figure 1C), we classified these clusters into seven major cell types, including T and NK cells (6 clusters), B cells (2 clusters), myeloid cells (2 clusters), mast cells (1 cluster), fibroblasts (3 clusters), endothelial cells (1 cluster), epithelial and malignant cells (3 clusters). To explore the cellular heterogeneity within these major cell lineages, we reclustered each of these major cell types using a finely tuned pipeline which had higher resolution to distinguish cells from similar subtypes or cells in different states within a single cell lineage (Figure S2, Methods).

### Significant expansion of regulatory T cell in gastric tumors

**Figure 2:**
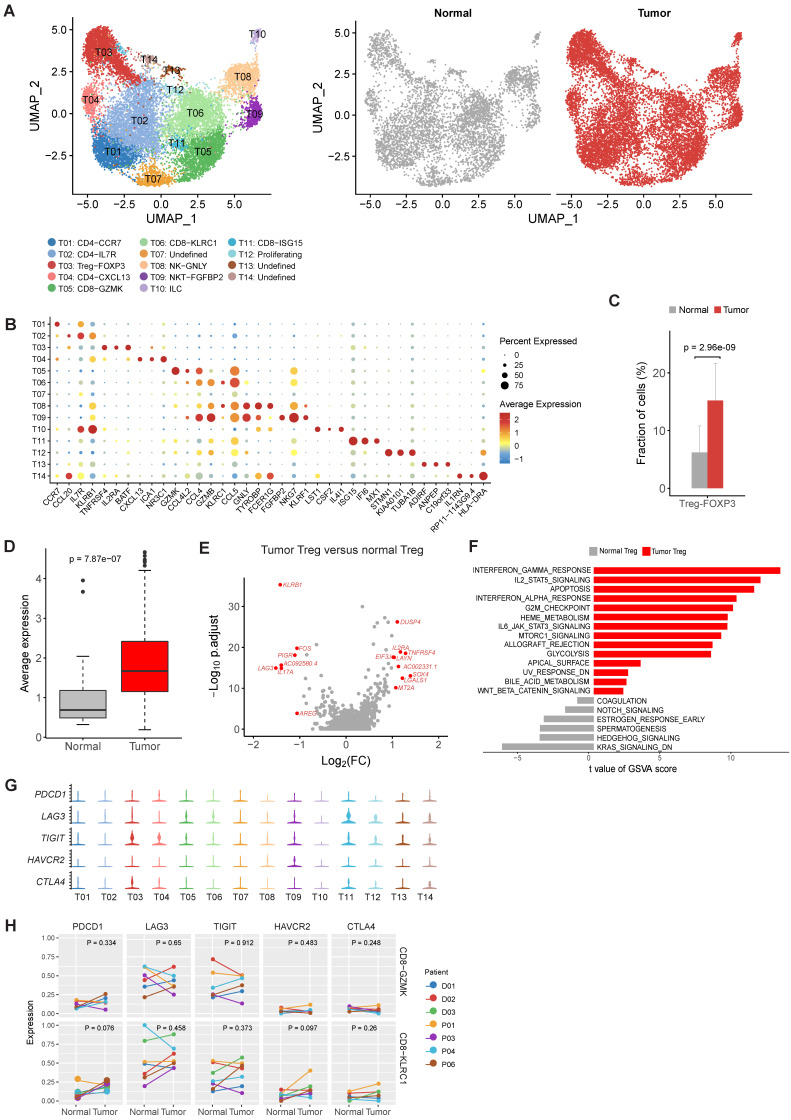
** The profile of T and NK cells in gastric cancer.** A, UMAP plots of T and NK cells, colored by cluster (left panel) and by tissue origin (right panel). B, Bubble plot of top differentially expressed genes for each T and NK cluster. C, The proportions of Tregs in tumor and normal samples. D, The average expression of Treg signature genes in tumor and normal samples of the gastric adenocarcinoma dataset in TCGA. E, Differentially expressed genes of Tregs between tumor and normal tissues. F, Pathways had increased activities in tumor Tregs estimated by GSVA. G, Violin plots of immune checkpoint genes in all T and NK clusters. The gene expression value of Y-axis ranges from 0-5. H, Differential analysis of exhaustion levels between tumor and normal samples.

T and NK cells were reclustered and 14 distinct clusters were identified (Figure 2A and Figure S3). Based on the expression of canonical marker genes and the top differentially expressed genes of each cluster (Figure 2B, Figure S3 and Table S2), we annotated these clusters as regulatory T cells, CD4+ T cells, CD8+ T cells, natural killer cells and innate lymphocyte cells (ILCs).

For CD4+ cell clusters (Figure 2B), T01 was characterized as native CD4+ T cells given the specific expression of CCR7 14. T02 was identified as helper T cells with increased expression of IL7R, CCL20 and GZMA (Figure 2B and Table S2) 14. T04 was classified as follicular help T cells based on specifically expressed CXCL13 14, and this cluster had increased expression of PDCD1 and TIGIT (Table S2), suggesting an exhausted state.

The relative proportions of Tregs (T03) in tumor samples were significantly higher in tumor samples comparing to adjacent normal samples (P = 2.96×10-9, Figure 2C), suggesting the expansion or recruitment of Tregs in gastric tumors. To further validate this observation, we calculated the average expression of 14 Treg signature genes (Table S3) among 328 gastric tumor samples and 32 non-malignant gastric samples in the bulk RNA-seq data of TCGA. Indeed, Treg signature genes showed consistent higher expression in tumor samples (P = 7.87×10-7, Figure 2D). While comparing with normal Tregs, tumor Tregs had increased expression of multiple genes related with immune suppression, including DUSP4, IL2RA, TNFRSF4, LAYN and LGALS1 (Figure 2E). Gene set variation analysis (GSVA) analysis revealed multiple immune response related pathways, namely IL2-STAT5 signaling, IL6-JAK-STAT3 signaling, allograph rejection, INF-alpha response and INF-gamma response, PI3K-AKT-MTOR signaling, KRAS signaling and glycolysis, were upregulated in tumor samples (Figure 2F). The increasing of Treg proportion in tumors, together with the upregulated expression of these genes and pathways, suggested the immunosuppressive nature of microenvironment in gastric tumors.

### No typical exhausted CD8+ T cell cluster was founded in gastric tumors

Next we investigated the two major clusters of CD8+ T cells, T05 and T06. T05 was characterized as effector memory CD8+ T cells (TEM) with high expression of GZMK and a set of cytotoxic genes 14 (Figure 2B). T06 was classified as tissue resident memory T cells (TRM) based on increased expression of KLRC1 and ITGA1/CD103 (Figure 2B). Intriguingly, we did not identified an typical exhausted CD8+ T cell cluster in our dataset, which had been frequently detected in various tumor types, such as colon cancer and non-small-cell lung cancer 15, 16. To confirm the absence of exhausted CD8+ T cells, we performed another reclustering on T and NK cells using an independent scRNA-seq dataset of gastric cancer published recently 12. Consistently, none of the CD8+ T cell clusters could be designated as exhausted CD8+ T cells in this independent dataset (Figure S4A-C). In addition, no differential expression of immune checkpoint genes in T05 and T06 was detected between tumor and normal samples (Figure 2G). These observations suggest that the exhaustion levels of cytotoxic CD8+ T cells are relatively low in primary gastric tumors.

We calculated the exhaustion scores for CD8+ T cells (the average expression values of PDCD1, LAG3, TIGIT, HAVCR2, CTLA4 in each cell), which was low among all CD8+ cell clusters (Figure S5). We also compared the exhaustion levels of CD8+ T cell clusters between tumor and normal samples. No significant increase of exhaustion levels was observed in tumor samples (Figure 2H). Therefore, CD8+ T cells in the gastric tumor did not have significant exhaustion.

### A tumor specific LAMP3+ dendritic cells (DCs) were identified in gastric tumors

**Figure 3:**
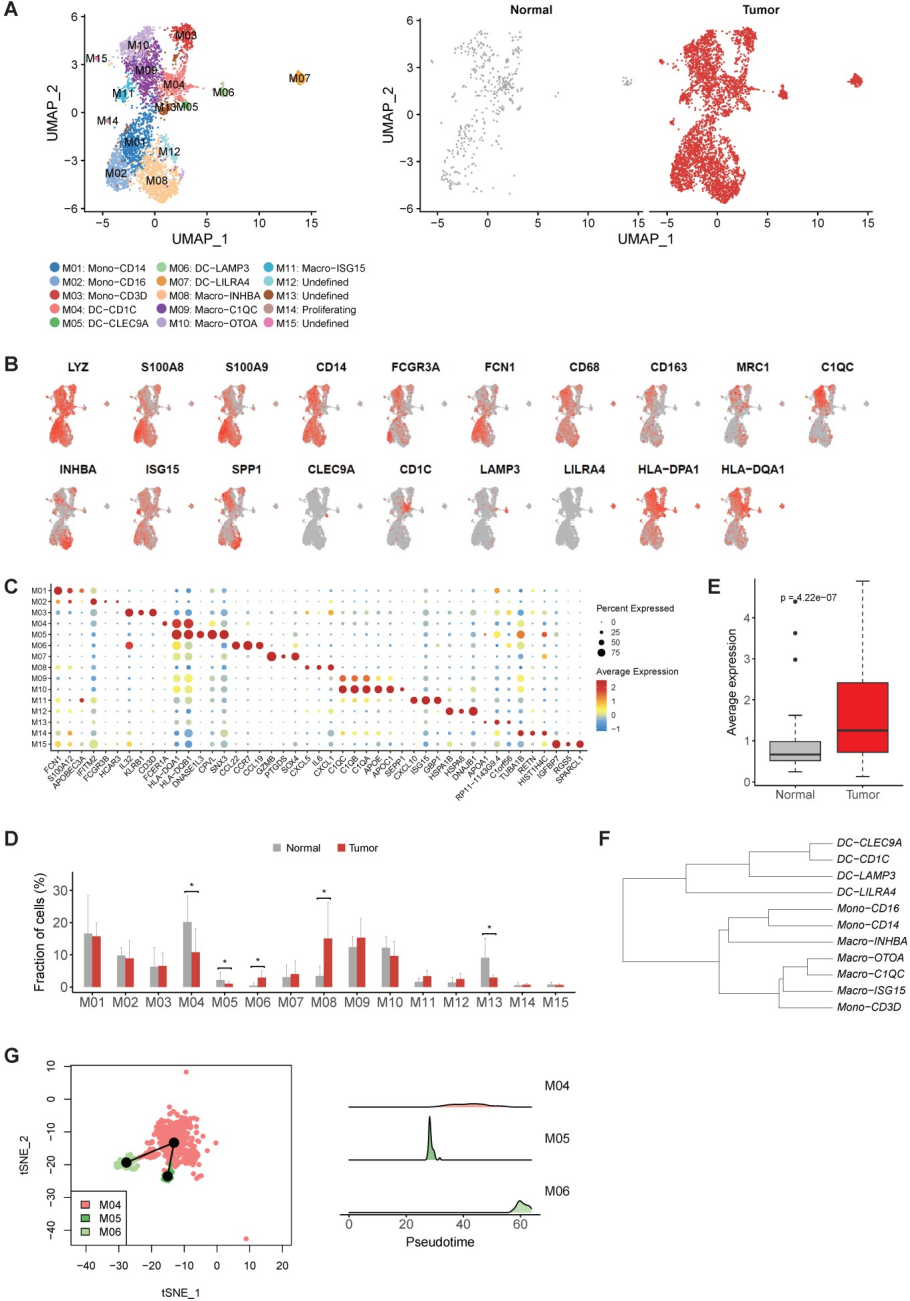
** The heterogenicity of myeloid cells in gastric cancer.** A, UMAP plots of myeloid cells, colored by cluster (left panel) and tissue origin (right panel). B, UMAP plots of marker genes of different subsets of myeloid cells. C, Bubble plot of top differentially expressed genes for each myeloid cell cluster. D, The proportions of each myeloid cell cluster in tumor and normal samples. Asterisks denote P value < 0.05 in the comparison of the proportions between tumor and normal samples. E, The average expression of signature genes of LAMP3+ DCs in TCGA gastric adenocarcinoma dataset. F, Dendrogram of different subsets of monocytes, DCs and macrophages in tumor samples. G, Trajectory analysis of three DC clusters by Slingshot. The density distribution of pseudotime for three DC clusters was plotted on the right panel.

Then we reconstructed myeloid cells clustering and identified 15 clusters (Figure 3A). M01-M03 were identified as monocytes based on the high expression of S100A8, S100A9 and FCN1 in these clusters (Figure 3B-C). M04-M07 were characterized as dendritic cells based on low expression CD14 and high expression of HLA-DR gene (Figure 3B-C). M08-M11 was identified as macrophage due to the high expression of CD68, CD163 and MRC1 in these clusters (Figure 3B-C). Remaining three unclassified clusters (M12, M13 and M15) were probably derived from cells of low quality or doublet cells.

The expression profiles of M01 (CD14highCD16-) and M02 (CD14+CD16high ) were similar to Mono1 and Mono 2 in human blood 17, i.e. classical monocytes and non-classical monocytes, respectively (Table S4). M03 expressed CD2, CD3D, IL32 and a number of cytotoxic genes (CCL5, TRAC, GZMA and GNLY) (Table S4), which was the unique characteristic of Mono4 cells in the same study 17. The consistency of all the three monocyte subtypes with cell subtypes in the blood reflects the infiltrating nature of monocytes in gastric tumors.

While M04 highly expressed CD1C, FCER1A and CLEC10A corresponding to cDC2 (Figure 3C and Table S4), M05 highly expressed CLEC9A representing cDC1 (Figure 3C and Table S4), and M07 was identified as plasmacytoid DC (pDC) by the specific expression of LILRA4 (Figure 3C and Table S4). However, we noticed M06, highly expressed LAMP3, CCL22 and CCL19, did not connect with any classical DC subtype (Figure 3C and Table S4). Unlike the previous three classical DC subtypes which were shared by both tumor and normal samples, almost all cells in M06 were derived from tumor samples, suggesting that this LAMP3+ DC subsets were strongly enriched in tumor samples (Figure 3D). Interestingly, the expression profile of this cluster was similar to the LAMP3+ DCs identified in hepatocellular carcinoma (Figure S6 and Table S5), which was also strongly enriched in tumor samples 18. Furthermore, the average expression of signature genes for LAMP3+ DCs was also much higher in the stomach tumor samples in TCGA dataset (Figure 3E and Table S5).

To explore the origin of LAMP3+ DCs in gastric tumors, we first built a dendrogram of myeloid clusters and found that LAMP3+ DCs were clustered with cDC1 and cDC2 (Figure 3F). Next, trajectory analysis of these three subsets revealed that LAMP3+ DCs potentially were developed from cDC2 and cDC1 in gastric tumors (Figure 3G). Lastly, SCENIC (Single-Cell Regulatory Network Inference and Clustering) analysis revealed that the activities of IRF1, IRF2, NFKB1 and NFKB2 were upregulated in LMAP3+ DCs (Figure S7), which indicates that IRF family and NF-kB are crucial regulators for DC differentiation and maturation 19.

### The heterogeneity of macrophages in gastric tumors

When focusing on macrophages, we observed M08 had high expression of INHBA, PTGS2 and a number of pro-inflammatory cytokines and chemokines, which was similar to the recently reported INHBA+ macrophages identified in esophageal carcinoma (Figure S8 and Table S4) 20. M09 and M10 were two clusters of C1QC+ macrophages, characterized by high expression of multiple C1Q genes and antigen presenting genes (Figure 3B). M11 had high expression of multiple interferon induced genes, such as ISG15, IFIT2 and IFIT3 (Figure 3C and Table S4). Among these four macrophage clusters, M08 was significantly enriched in the tumor samples (Figure 3D), thus was designated as tumor associated macrophages (TAMs).

**Figure 4:**
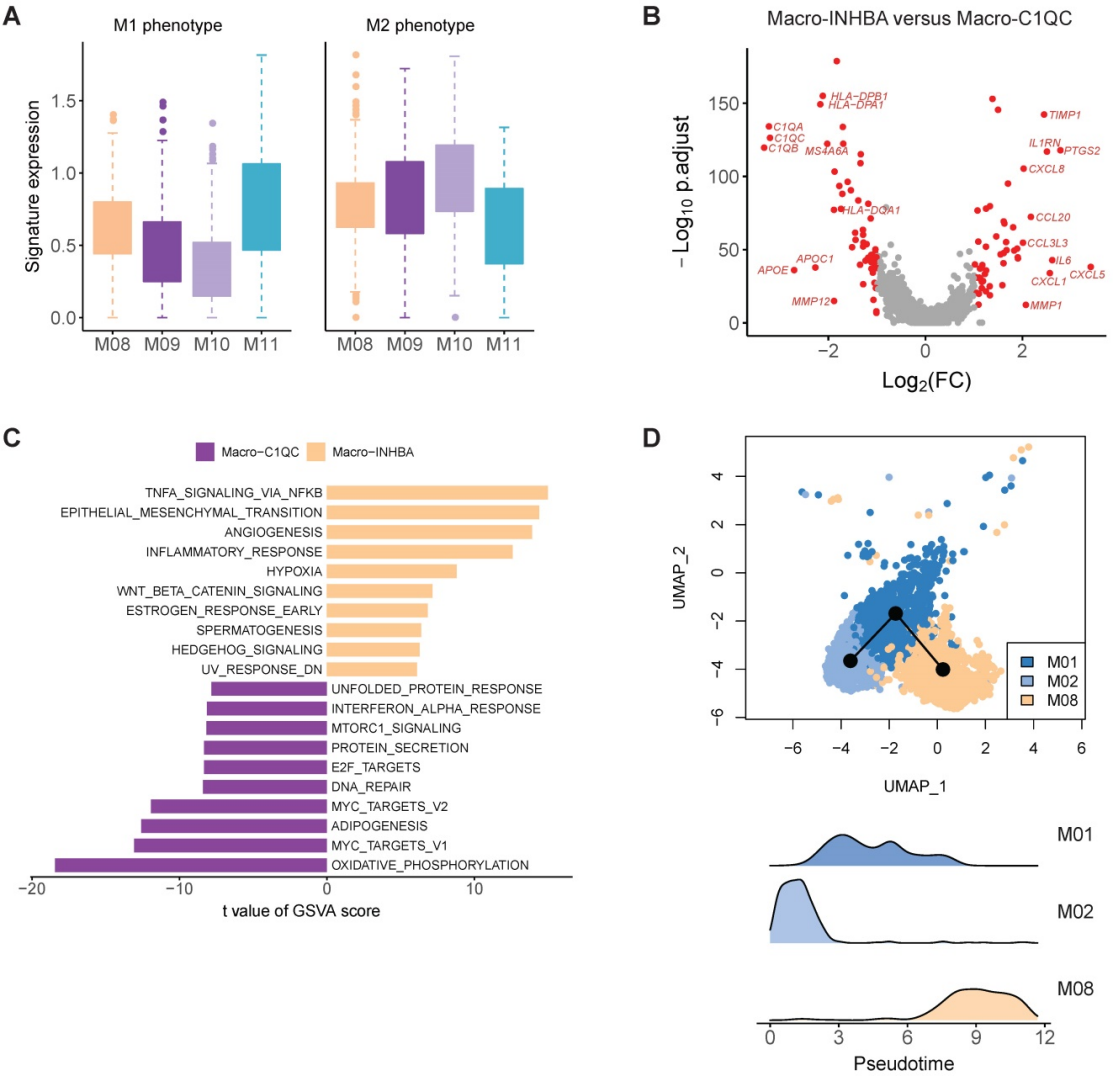
** The molecular features of macrophage in gastric cancer.** A, Boxplots showing the average expression of M1 and M2 signature genes in four macrophage clusters. B, Differentially expressed genes between Macro-INHBA and Macro-C1QC. C, Pathways had high activities in Macro-INHBA and C1QC+ Macro-C1QC by GSVA. D, Trajectory analysis of M01, M02 and M08 by Slingshot. The density distribution of pseudotime for the three clusters was plotted on the bottom panel.

Macrophages are usually classified into pro-inflammatory M1 and anti-inflammatory M2 class 20. To test whether the macrophage subpopulations identified here fit the classical M1/M2 model, we evaluated the expression of the classical M1 and M2 signatures for these macrophage subtypes. M1 and M2 signature genes were co-expressed by all these subpopulations. The INHBA+ cluster (M08) and ISG15+ cluster (M11) exhibited higher M1 signatures, while the C1QC+ cluster (M9 and M10) were higher for M2 signatures (Figure 4A). This analysis suggests that *in vivo* polarization of macrophage in gastric tumors could not be explained by the M1/M2 model.

In the comparison of gene expression levels of TAMs and C1QC+ macrophages, we identified 49 upregulated genes (fold change > 2, FDR < 0.05) in INHBA+ macrophages, including multiple chemokines, IL6, PTGS2, IL1RN and TIMP1 (Figure 4B). GSVA analysis of hallmark pathways revealed there were increased activities of WNT beta catenin signaling, angiogenesis, hedgehog signaling, epithelial mesenchymal transition, and IL10 signaling in this subtype, while C1QC+ macrophages were upregulated in MHC class II antigen presentation (Figure 4C). INHBA+ TAM and monocytes were located in a single branch in the dendrogram, implying that this cluster probably was originated from infiltrated monocytes in tumor regions (Figure 3F). Trajectory analysis supports that this TAMs (M08) could develop from monocytes (Figure 4D). SCENIC analysis revealed that the activities of multiple transcription factors were specifically upregulated in INHBA+ TAM, including RELB, NFKB1, NFKB2 and etc. (Figure S9).

### Fibroblasts play crucial roles in neovasculation and tumor development

**Figure 5:**
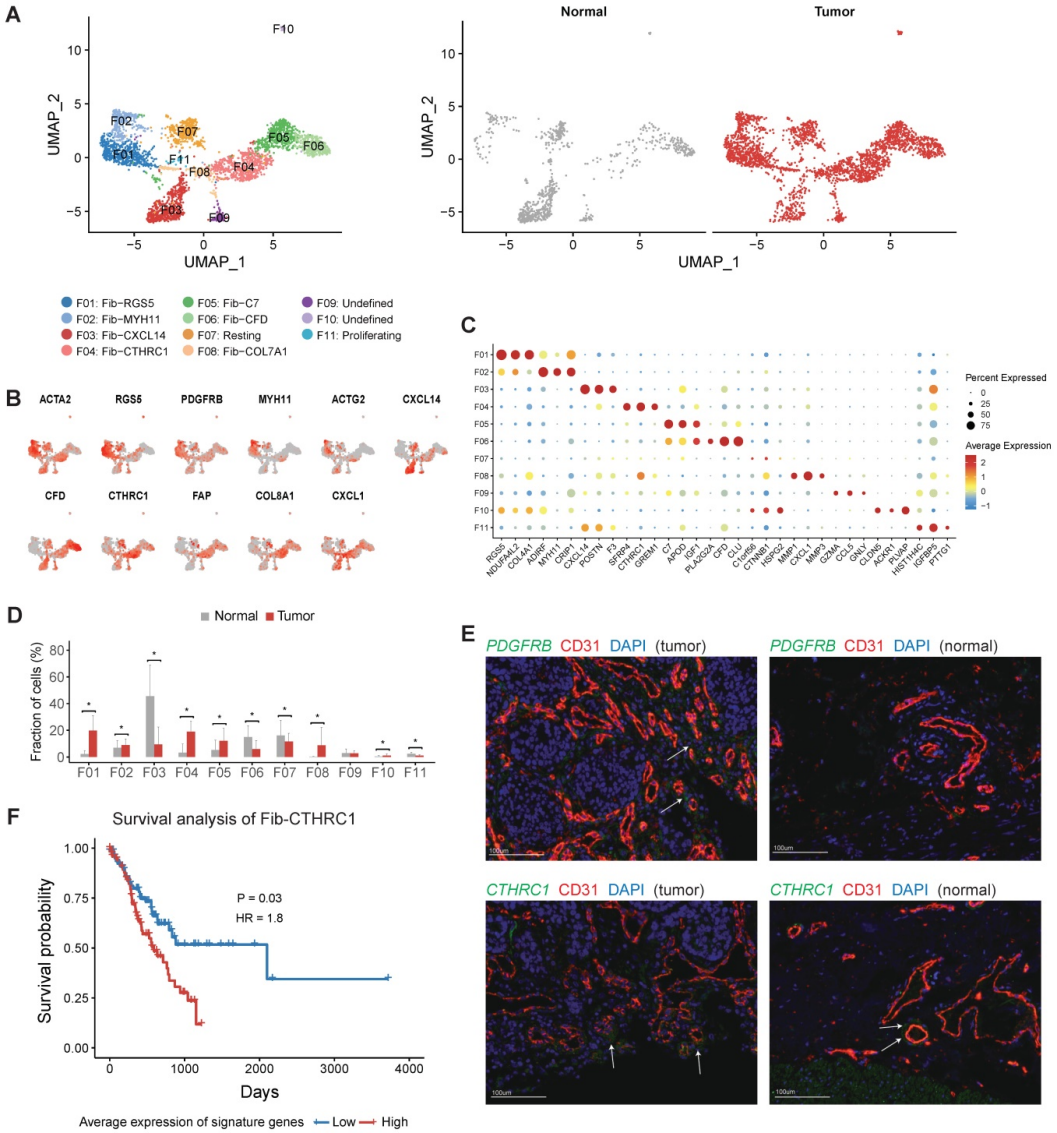
** The profile of fibroblasts in gastric cancer.** A, UMAP plots of fibroblast cells, colored by cluster (left panel) and tissue origin (right panel). B, UMAP plots of marker genes for different subsets of fibroblasts. C, Bubble plot of top differentially expressed genes for each fibroblast cluster. D, The proportions of each fibroblast cluster in tumor and normal samples. Asterisks indicate P value < 0.05 in the comparison of the proportions between tumor and normal samples. E, Immunofluorescence staining of PDGFRB (top panel) and CTHRC1 (bottom panel) together with CD31 (vascular endothelial cells) and DAPI (nuclei) (100 µm). F, Kaplan-Meier survival curves showed the gastric cancer patients with high expression of Fib-CTHRC1 signature genes had poor survival in TCGA datasets.

Next, we aimed to explore the heterogeneity of fibroblasts in gastric cancer. Fibroblasts are thought to be a highly plastic cell population in the TME, but no consensuses were achieved for the definition of fibroblast subtypes and CAFs in gastric cancer. Here, we obtained 11 fibroblast clusters in total after the reclustering of the 3,467 fibroblast cells and designated three of them as CAF (Figure 5A). The expression of collagens, MMPs, cytokines and chemokines varied among different fibroblast clusters (Figure S10). For example, COL7A1 was specifically expressed in F08, and COL8A1 was highly expressed in F04 but not in other clusters (Figure S10A).

Both F01 and F02 expressed ACTA2 and multiple genes were related with muscle contraction (including ACTG2, MYH11 and PLN) (Figure 5B and Table S6). F01 could be annotated as pericytes referring to the high expression of RGS5 and PDGFRB in this cluster (Figure 5B-C). The relative abundance of pericytes was much higher in tumor samples (P < 0.05, Figure 5D). Immunofluorescence staining of PDGFRB showed that pericytes had a perivascular location and confirmed that pericytes were enriched in tumor samples (Figure 5E). Increased expression of several angiogenic factors (including ANGPT2, CAV1, NOTCH3, PDGFA, EPAS1 and THY1) was observed in this cluster (Figure S10B). Notably, gastric cancer patients with higher expression of PDGFRB had poor prognosis in TCGA (Figure S11). Therefore, this cluster plays crucial roles in the neovasculation of gastric tumors. SCENIC analysis further showed that LRRFIP1, ETS1, EPAS2, MEF2C and SSRP1 were upregulated in pericytes (Figure S12).

F03 was the major fibroblast subset in normal samples (Figure 5D), marked by high expression of CXCL14, POSTN, F3, PDGFRA and SOX6 (Table S6). The expression signature of this cluster was similar to the S2 subset of mesenchymal cells in human colons, which was in close proximity to the epithelial monolayer of colon and was thought to play some roles in the maintenance of epithelial homeostasis 21. In this study we observed significant decrease of this subset in tumor tissues, which could reflect the dysfunction of epithelial barrier in the tumors.

F06 was mainly derived from normal samples, marked by high expression of CFD, CLU, COL14A1 and PI16 (Table S6). COL14A1+ fibroblasts had been identified in single-cell studies of multiple human tissues, including human lung and colon tissues 21-23, indicating this cluster may represent a common subtype of fibroblast in multiple tissues. A set of ECM molecules (DCN, DPT, FBLN1, FBLN2, GSN and TNXB) were highly expressed in this cluster. In addition, we found multiple complement factors (Figure 5C and Table S6) were highly expressed in this cluster, implying that this subset of fibroblasts plays some roles in innate immune defense.

F04, F05 and F08 were significantly enriched in tumor samples, thus were denoted as three subsets of cancer associated fibroblasts (CAFs) (Figure 5D). F04 specifically expressed CTHRC1 (Figure 5C and Table S7), which was over-expressed in gastric tumors and associated with poor prognosis 24. It has been reported that CTHRC1 was associated with tumor progression and metastasis in multiple tumor types 24-29. Immunofluorescence staining of CTHRC1 showed the location of CTHRC1+ cells was endothelial cell surrounding in gastric tumors (Figure 5E). FAP, a canonical marker of CAF, had highest expression in this cluster (Figure 5B). In the TCGA dataset, high expression of signature genes of Fib-CTHRC1 had poor prognosis (Figure 5F, Methods). GSVA analysis revealed that this cluster had high activities in ECM remodeling related pathways, including "Elastic fiber formation", "Activation of matrix metalloproteinases" and "Collagen degradation" (Figure S13).

F05 was a COL14A1+ fibroblast cluster, characterized by high expression of C7 and APOD (Figure 5C). F08 specifically expressed MMP1, MMP3 and MMP9 (Figure S10). Transcription factor TWIST1 was highly expressed in F08 (Figure S10), which has been validated as a key regulator of cancer-associated fibroblast 30.

### Tumor endothelial cells showed high activity of angiogenesis

**Figure 6:**
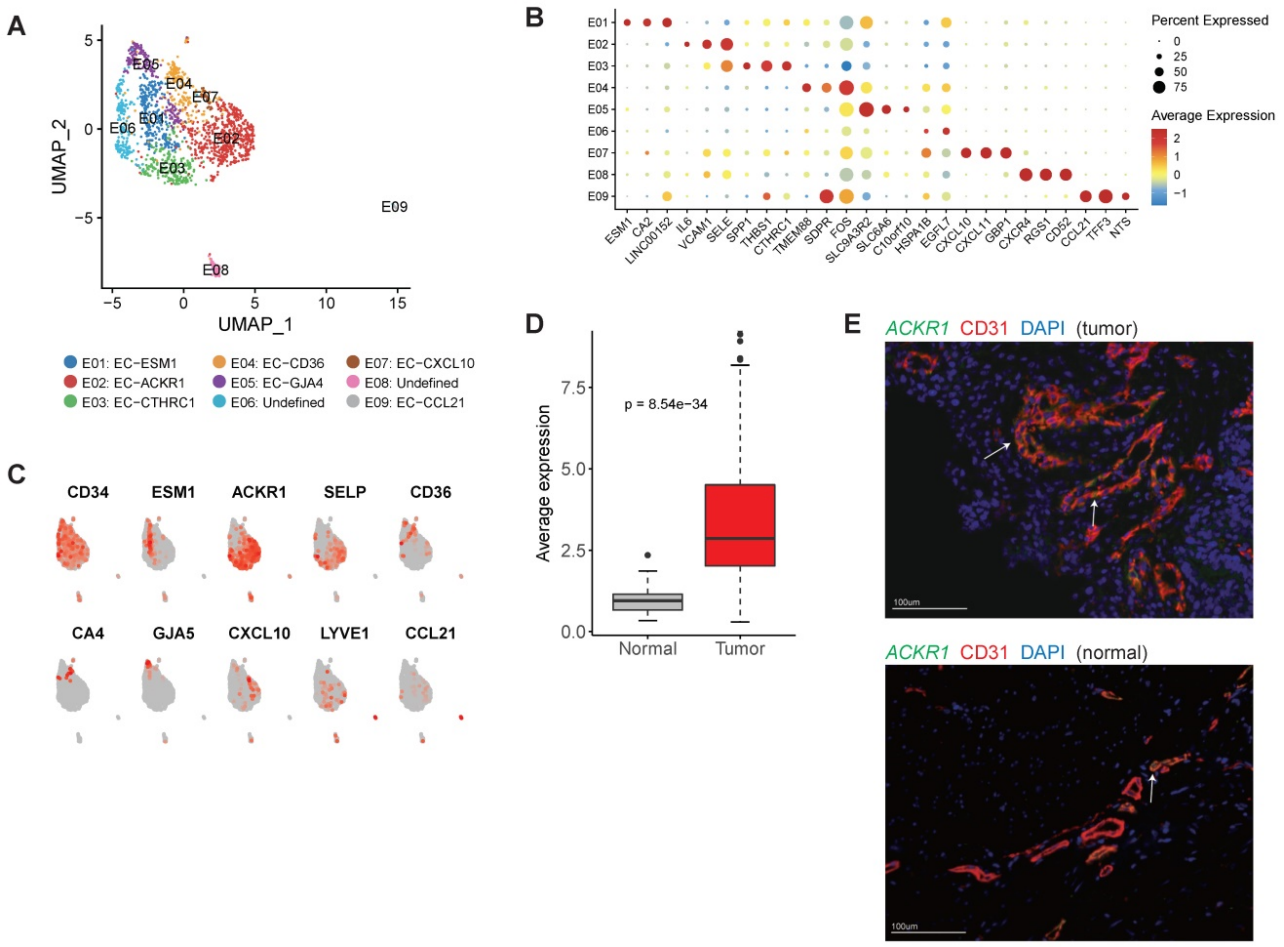
** The cellular composition of endothelial cells in gastric cance**r. A, UMAP plots of endothelial cells, colored by cluster (left panel) and tissue origin (right panel). B, Bubble plots of top differentially expressed genes for endothelial cell clusters. C, UMAP plots of CD34 and marker genes for different subsets of endothelial cells. D, The average expression of signature genes of EC-ESM1 in TCGA gastric adenocarcinoma dataset. E, Immunofluorescence staining of ACKR1 together with CD31 (vascular endothelial cells) and DAPI (nuclei).

Overall, we detected 1,873 endothelial cells (ECs) in this study and most of them (88.2%) were derived from the tumor samples. Reclustering of tumor endothelial cells revealed nine distinct clusters (Figure 6A).

E01 was tip-like cells with specific expression of tip cell markers (including ESM1, KDR and PDGFB) were observed in this cluster (Figure 6B-C and Table S8). Multiple VEGF receptors were expressed in this cluster (Table S8), in line with the essential roles that tip cells played in angiogenesis. We identified 3 signature genes for this cluster, then compared the average expression signature genes between gastric tumor samples and non-malignant gastric samples in TCGA datasets (Table S9). In TCGA, gastric tumor samples had much higher expression of these signature genes, indicating angiogenesis was very active in gastric cancer (Figure 6D). GO enrichment of upregulated genes showed that "angiogenesis" and "leukocyte chemotaxis" were enriched in this cluster (Figure S14).

E02 and E03 had high expression of ACKR1 and SELP (Figure 6C), thus could be designated as venous ECs 31. Immunofluorescence staining showed that ACKR1 were highly expressed in tumor ECs (Figure 6E). In the TCGA dataset, gastric cancer patients with high expression of ACKR1 gene had poor prognosis (Figure S15). E04 had high expression of CD36 and CA4 (Figure 6C), representing capillary ECs 32. E05 had high expression of multiple markers of arterial ECs (GJA5, GJA4, SEMA3G and HEY1) (Table S8). E07 expressed a number of interferon induced genes (such as CXCL10, CXCL11, ISG15 and IFIT3) (Table S8). E09 represented lymphatic EC as LYVE1 and CCL21 were specifically expressed in this cluster (Figure 6C).

### Enhanced interplay of endothelial and fibroblast in tumor angiogenesis

**Figure 7:**
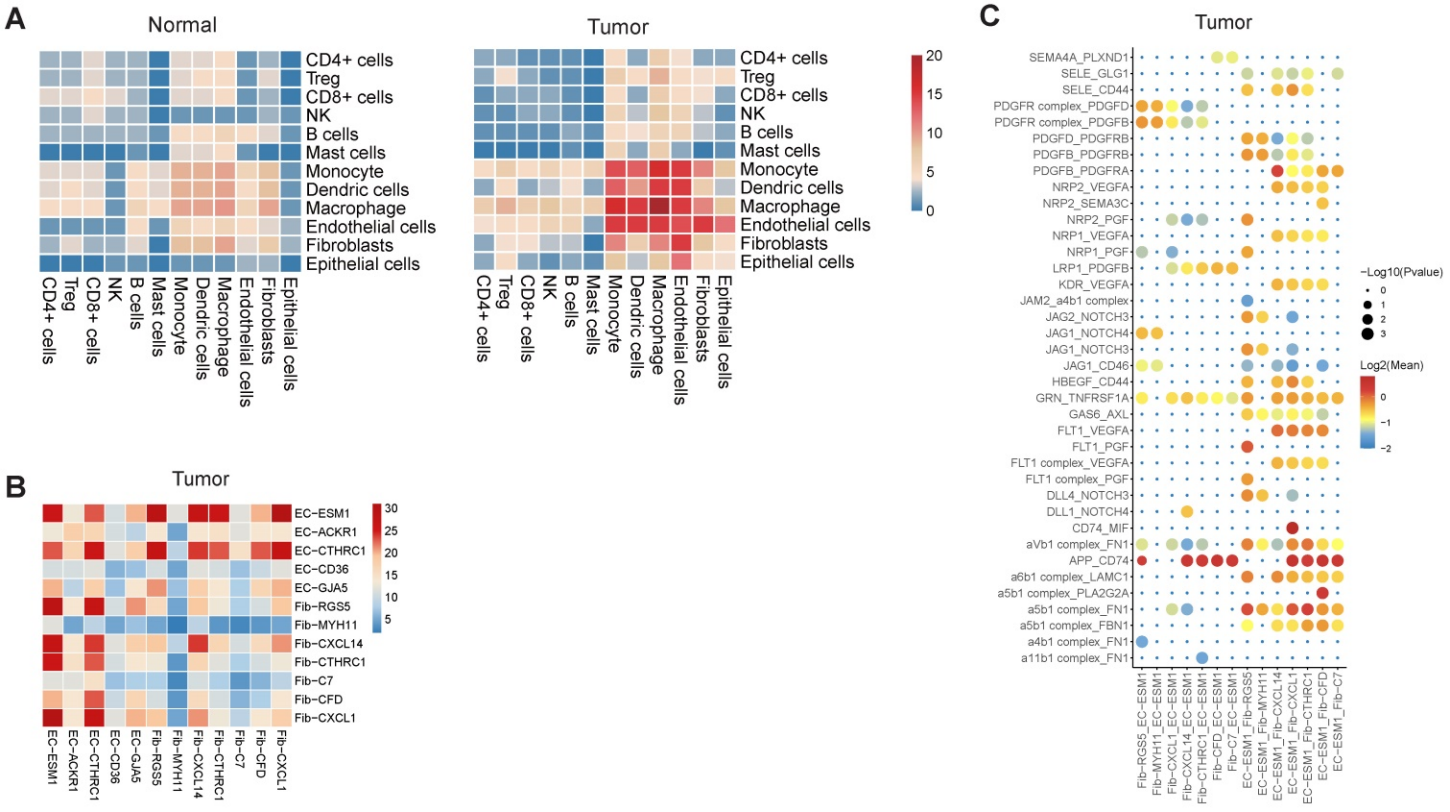
** Cell-cell interactions in gastric cancer**. A, Heatmap representing the number of predicted ligand-receptor pairs between different cell types in tumor and normal samples. B, Heatmap representing the number of predicted ligand and receptor pairs between different subsets of endothelial cells and fibroblasts in tumor samples. C, Dot plot of predicted ligand-receptor interactions between different subsets of endothelial cells and fibroblasts in tumor samples.

Using CellPhoneDB2, we identified potential cell-cell interactions mediated by various ligand and receptor pairs in tumor and normal samples (See Methods) 33. In normal samples, cell-cell interactions were slightly enriched in three classes of myeloid (Figure 7A), in line with the essential roles that myeloid cells played in the maintenance of the tissue homeostasis. In tumor samples, we observed enhanced interactions between endothelial cells and multiple cell types, including fibroblast, monocytes, macrophages and DC (Figure 7A). For example, there were only 3 predicted interactions between endothelial cells and fibroblasts in normal samples, but the number was 15 in tumor samples. The tight interaction between endothelial cells and fibroblast implied that fibroblast was closely implicated in tumor angiogenesis and the maintenance of tumor vasculature.

When inspecting the cell-cell interactions for different subsets of endothelial cells and fibroblasts, we found EC-ESM1 (tip-like endothelial cells) had strong interactions with four subsets of fibroblast (F01-F04) (Figure 7B). The interaction of EC-ESM1 and these four subsets of fibroblasts was mainly mediated by PGF, VEGFA, PDGF genes and their receptors (Figure 7C), which were the known driving factors for angiogenesis. Interestingly, the interaction of FLT1 and PGF was only observed in EC-ESM1 and Fib-RGS5, but not in other pairs of endothelial and fibroblast clusters. Taken together, our analysis showed that the enhanced interplay of endothelial and fibroblast was the fundamental change of cell-cell interactions in gastric cancer.

### The replication analysis of three previous studies

Three published scRNA-seq datasets (PMID 32532891, 34385296 and 34933901) 12, 34, 35 were used to verify that the cells subpopulations identified in our datasets could represent the cellular heterozygosity of gastric cancer. As shown in Figure S16, UMAP plot of T cell subpopulations identified in PMID 32532891 (Figure S16A), PMID 34385296 (Figure S16B) and PMID 34933901 (Figure S16C), where regulatory T cells, CD4+ T cells, CD8+ T cells and natural killer cells were found in all three studies. Clustering of myeloid cells from these published single cell datasets of gastric cancer was showed in Figure S17, and 15 clusters were found in three datasets. In Figure S18 and Figure S19, fibroblast and endothelial cells with from gastric cancer were analyzed, and the majority of the subtypes were similar to our findings.

## Discussion

In this study we generated a complete single-cell atlas of immune and stromal cells for gastric cancer by scRNA-seq. Using a finely tuned clustering method, we unveiled the cellular heterozygosity of T and NK cells, myeloid cells, fibroblasts and endothelial cells in gastric cancer.

For T and NK cells, we found there was significant expansion of Tregs in tumor samples. The cytotoxic activity of CD8+ T cells could be rendered ineffective primarily by the suppression of Tregs, defined by poor effector function, sustaining expression of inhibitory receptors and a unique transcriptional state 16, 36. Therefore, a deep understanding of the mechanisms and pathways leading to the accumulation of Tregs in cancer will provide better strategies to orchestrate the immune system to eradicate cancers. The increased expression of multiple immune suppression genes was observed in tumor Tregs. For example, LAYN, linked to the suppressive function of tumor Treg and exhausted CD8+ T cells, was recently reported to be highly expressed in Tregs isolated from lung, colon and liver cancers 22. LGALS1 contributes to the immune heterogeneity and immunosuppression in glioma 37. DUSP4 is important not only for both innate and adaptive immune responses, but also for metabolic homeostasis 38. IL2RA, highly expressed in Treg cells, has strong suppressive activity^39^. The upregulation of these genes suggest that microenvironment of gastric tumors was more immunosuppressive than normal samples. Surprisingly, we did not obtain a separate cluster of typical exhausted CD8+ T cells. The exhaustion markers PDCD1, CTLA4, HAVCR2, LAG-3, and TIGIT were expressed at low levels in CD8+ T cells, which implied the benefit of immunotherapy would be limited for gastric cancer patients 16, 40.

Macrophage metabolism has been tightly associated with distinct activation phenotypes within the range of M1-like and M2-like types 41. However, in this study, M1-like and M2-like TAM signature genes were co-expressed by all macrophage subpopulations, suggesting the polarization of TAM in gastric cancers could not be explained by the fully M1/M2 (or alternatively activated) macrophages model. This is consistent with a previous study 11, indicating that tumor-associated macrophages have a spectrum-like level of subtypes in microenvironment. In this study, we identified a subset of macrophage as tumor associated macrophage (M08: Macro-INHBA), which probably were monocyte derived. This subset showed high activity of angiogenesis, hedgehog signaling, epithelial mesenchymal transition, NF-κB pathway, and IL10 signaling, suggesting it had essential roles in the remodeling of cancer immunity and progression. In addition, a published study indicated that INHBA over-expression promotes cell proliferation and may be epigenetically regulated in esophageal adenocarcinoma 42.

In previous studies, CAF is characterized by increased expression of myofibroblast markers, there is no consensus on what distinguishes quiescent fibroblasts, myofibroblasts and CAFs 43, 44. Increasing evidence has shown that CAFs mediate chemotherapy resistance in several tumors by releasing paracrine signals such as cytokines, exosomes and metabolites 45, 46. In this study, we found pericytes were enriched in gastric tumors and identified three subsets of fibroblast as CAFs. One of these subsets (labeled as Fib-CTHRC1) was represented by high expression of CTHRC1. CTHRC1 is upregulated by promoter demethylation and transforming growth factor-β1 and may be associated with metastasis in human gastric cancer 47. We demonstrated that high expression of signature genes of F04 was associated with poor survival in gastric cancer. Therefore, targeting CAFs may be an innovative strategy that may synergize with the standard antitumor approaches and serve as a more effective combination therapy for gastric cancer.

Abnormal vessel growth and function are hallmarks of cancer, contributing to disease progression 48. Therapeutic approaches to block the vascular supply have reached the clinic, but the limited efficacy due to cancer resistance poses unresolved challenges 49. In this study, we observed the interactions of endothelial cells with multiple cell types were enhanced in tumor samples. Specifically, there was strong interaction between tip-like ECs and CAFs, mediated by PGF, VEGFA, PDGF and their receptors.

In addition, for most cell sub-populations identified in our datasets there is a counterpart in the three published scRNA-seq datasets of gastric cancer. All these findings indicated that the cell sub-populations detected in our datasets could represent the cellular heterozigosity in the micro-environment of gastric cancer.

This study has three main limitations. As a small number of samples were collected in our study, increasing the cohort size will help to address the influence of the TME in gastric cancer, further replicate and validate the generalizability of our findings. Secondly, we did not consider spatial context, which may be affected by the dissociation process and could be addressed by dual single-cell proteomics and transcriptomics. Thirdly, for the first time we demonstrated the profiling of stromal cells and immune cells in gastric cancer, rather than solely epithelial cells. Fourthly, it's difficult to describe the cellular heterogeneity of tumor cells, as well as the interaction between tumor epithelial cells and other cells because of the length limit in this manuscript. We will discuss it in our future study.

Taken together, our single-cell atlas of gastric cancer could shed more light on the potential solutions on efficiently inhibiting tumor angiogenesis and tumor cell proliferation and invasion.

## Methods

### Patient recruitment and ethical approval

Nine patients with histologically confirmed proximal (n = 6) or distal (n = 3) gastric adenocarcinoma were enrolled in this study, and normal stomach tissues from these patients were collected as control samples. All patients were treatment-naïve and their clinical characteristics are summarized in Table S1. All clinical samples were collected from the Center for Cancer/Cancer Hospital, Chinese Academy of Medical Sciences and Peking Union Medical College from 2017 to 2018. Written informed consent was obtained from all participants enrolled in this study, and ethical approval was obtained from the following institutional review boards in accordance with the Declaration of Helsinki: National Cancer Center/National Clinical Research Center for Cancer/Cancer Hospital, Chinese Academy of Medical Sciences and Peking Union Medical College. Approval number: 17-156/1412. Issued date: 2017-09-14.

### Sample processing and library construction for scRNA-seq

Fresh gastric tumor and adjacent tissues were cut into approximately 1 mm3 pieces in RPMI-1640 medium (Invitrogen) with 10% fetal bovine serum (FBS; ScienCell) and enzymatically digested with a MACS tumor dissociation kit (Miltenyi Biotec) for 30 min on a rotor at 37°C, according to the manufacturer's instructions. After filtration with a 70 μm Cell-Strainer (BD) in RPMI-1640 medium (Invitrogen), the suspended cells were centrifuged at 400× g for 5 min. After removing the supernatant, the pelleted cells were suspended in red blood cell lysis buffer (Solarbio) and incubated on ice for 2 min to lyse red blood cells. The cell pellets were resuspended in sorting buffer (PBS supplemented with 2% FBS) after washing twice with PBS (Invitrogen).

The single cell suspensions were stained with 7-AAD Viability Staining Solution (Cat# 00-6993-50, eBiocience) for flow cytometry (FACS), performed on a BD Aria III instrument. Based on FACS analysis, 1 × 105 living cells were sorted into 1.5 ml tubes with sorting buffer, and counted manually under the microscope. Then, single cells were processed with the GemCode Single Cell Platform using the 3' GemCode Gel Bead, Chip and Library Kits (10 x Genomics) as per the manufacturer's protocol. The loaded cell numbers were 10,000 for each sample. The cells were then partitioned into Gel Beads in Emulsion in the GemCode instrument, where the cells were lysed and barcodes were ligated via reverse transcription; then, the RNA was amplified and sheared, and 3' adaptors and sample indexes were ligated. The libraries were sequenced on an Illumina HiSeq 4000 with paired-ends 150bp sequencing strategy.

### Single cell data processing and clustering

Raw gene expression matrix was generated for each sample using CellRanger (v2.0.2). Cells that had less than 200 expressed genes or more than 7,000 expressed genes were removed. Cells in which the fraction of mitochondrial genes exceeded 10% also were removed. Scrublet (v0.1) was used to remove doublet cells. The UMI count per gene were normalized by the total UMI count in each cell and log transformed with the NormalizedData function in Seurat 50, using 10000 as the scale factor. The effects of the number of detected UMIs, the fraction of mitochondrial genes and cell cycles on the gene expression values were corrected by regression using the ScaleData function in Seurat.

Before the clustering, we first applied Canonical correlation analysis (CCA) implemented in Seurat to correct the batch effects among the experiments, and integrate the gene expression matrix of all samples into a whole matrix. To identify the major cell types in our dataset, we first selected the variably expressed genes using the FindVariableGenes function in Seurat, requiring the average expression was between 0.05 and 5 and the dispersion was no less than 0.5. Next, we performed principal component analysis to reduce the dimensionality. The top 20 PCs were selected for cell clustering after the inspection of the elbow plot. The FindClusters function was used to cluster all cells from both tumor and normal samples at resolution 0.4. Finally, we got the tSNE visualization using the RunTSNE functions in Seurat. We also performed cell clustering using Harmony (v1.0) and compared the results of two clustering methods. The clustering results by Seurat had high agreement with the results by Harmony. In the analysis above, Seurat v3.2.2 was used.

### Reclustering of major cell types

To identify subtypes or cells in different states within a major cell type, we used a two round clustering strategy. Firstly, cells belonging to a cell type were extracted from the normalized gene expression matrix of each sample and a combined gene expression matrix of all samples was prepared. Like we did on the whole dataset, variably expressed genes were identified by the FindVariableGenes function in Seurat using the same parameters. After PCA analysis, we selected top PCs based on the elbow plot and performed clustering analysis using Harmony. To improve the resolution of cell clustering, we applied two-way ANOVA to identify genes in the variably expressed genes whose variance of expression were mainly derived from samples rather than cell clusters (the fraction of variance explained by samples in the total variance explained by samples and clusters > 0.9). After the removal of these genes from the list of variably expressed genes, we performed the second round of clustering. For each cell type, cell clustering was conducted at multiple resolutions (0.4, 0.6 and 0.8) and we finally chose the resolution which could give us the better recovery of the known subtypes or states within a cell type.

We then evaluated the robustness of reclustering using a resampling approach. Explicitly, we sampled 75% cells from our dataset at random, and then clustered them with same set of parameters and selected features. For each cluster, we calculated the fraction of the sampled cells that still clustered into a single cluster in the resampling as a measurement of cluster robustness. Most subclusters identified in our dataset are highly robust at 30 times of resampling (Figure S20).

### Comparison of cell cluster abundance between tumor and normal tissues

To access whether a specific cell cluster in a major cell type was significantly enriched in the tumor samples, we model the number of cells in a cluster as a random variable of a Poisson process. The condition (tumor or non-tumor) was provided as covariate and the total number of cells was provided as an offset variable. glm function in R package was used to fit the model 51. The significance of coefficient was evaluated using Wald test.

### Differential gene expression analysis

The MAST (Model-based Analysis of Single-cell Transcriptomics) method implemented in FindMarkers function in Seurat package was used to identify differently expressed genes between two subsets or clusters of cells. Genes with expression percent > 10%, fold change > 2 and Benjamini-Hochberg adjusted p < 0.05 were identified as differently expressed genes.

### Identification of signature genes

Signature genes for each cluster would have high expression in this cluster but not in other clusters of a cell type, also have specific expression in the current cell type but not in other cell types. To identify signature genes for a cluster in a cell type, we first identified differently expressed genes for this cluster using FindAllMarkers function in Seurat, and then calculated a cluster specificity score and a cell type specificity score for each differently expressed genes.

Cluster specificity score was defined as:



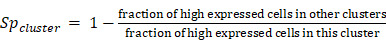



Here, high expressed cells was defined as cells whose expression were greater than the 1/4 quantile of the expression values in this cluster (ignore zero values). If a gene was only expressed in this cluster but not in other clusters in a cell type, cluster specificity score would be 1. If the fraction of high expressed cells other clusters in a cell type was equal to or higher than the fraction in this cluster, the cluster specificity would be 0.

Cell type specificity score was defined as:



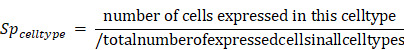



Here, a cell was taken as expressed if the expression value was greater than the 5th percentile of the expression values of all cells (ignore zero expression).

Finally, we calculated a combined score which was the geometric average of the cluster specificity score and the cell type specificity score:



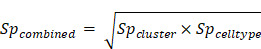



Using this combined score and a cutoff value of 0.7, we identified signature genes for regulatory T cells, LAMP3+ DC, INHBA+ macrophages and other cell clusters in our dataset. For regulatory T cells, a set of well-defined marker genes (including IL2RA, BATF, FOXP3, TIGIT, CTLA4 and ICOS) were identified as signature genes, indicating that this quantitative method was effective to identify the signature genes for a single cell cluster from single cell sequencing data.

### GSVA

The gene set variation analysis (GSVA) were performed on the hallmark pathways or canonical pathways collected in the molecular signature database (V7.0) 52, 53, and GSVA scores were obtained using the GSVA package (v1.34.0) 54. To assess the differential pathway activities among cell clusters, we used LIMMA package (v3.42.2) to contrast the activity scores for each cell based on a generalized linear model with the patient of origin as a categorical variable.

### Dendrogram

To explore how the clusters or subsets of a single cell type related with each other. We built dendrogram based on the similarities of their transcriptome, referring to the method used in Cheng et al 20. Explicitly, we identified variably expressed genes for a cell type and calculated their mean expression for each cluster. For each pair of clusters, we computed Pearson correlation coefficient of mean expression of variably expressed genes, and defined the distance between the two clusters as (1 - Pearson correlation coefficient)/2. Dendrogram was constructed using APE package (version 5.3).

### Trajectory analysis

Before trajectory analysis, cell cycle effects in major cell types were regressed out using the Seurat package 55. Slingshot (v1.4.0) was used to do trajectory construction by setting dimensionality reduction method as PCA and other parameters as default. Then lineages and pseudotime tendency within different cell clusters were visualized in graphs.

### Cell-cell interaction analysis

We used CellPhoneDB (v.1.1.0) 33 to detect the pairwise interactions between cell clusters. Only receptors and ligands whose expression was detected in more than 25% of cells were included in this analysis. The significance of a ligand and receptor pair in each cell-cell interaction was evaluated by 1,000 random permutations of the cell types. For each permutation, the total mean of the average receptor expression level and the average ligand expression level is calculated, and a null distribution is derived for each ligand-receptor pair. For the multi-subunit heteromeric complexes, the member of the complex with the minimum average expression is used for calculating the mean. An empirical P value is calculated from the proportion of the means which are 'as or more extreme' than the actual mean. The cell-cell interaction landscape was generated using Cytoscape (version 3.6.1)^56^. The network output was a circular layout and was adjusted manually.

### TCGA data analysis

The TCGA gastric adenocarcinoma dataset was used to evaluate the prognostic effect of a single gene or a set of genes (such as Treg signature genes). Gene expression data were downloaded from UCSC Xena (http://xena.ucsc.edu/), and the clinical data were downloaded from the Genomic Data Commons Data Portal (https://gdc-portal.nci.nih.gov/). The TPM values were normalized by the average expression of a gene in normal samples. For each signature gene set, the average TPM value of the selected genes was calculated for all samples. Samples whose average expression at the top 25% were defined as the high expression group, whereas samples whose average expression at the bottom 25% were defined as the low expression group. We performed multivariate analyses using the Cox proportional hazards model to correct clinical covariates including age, sex, tumor stage and gene expression group (high or low) for all survival analyses in our study (R Package survival, version 3.2-7). Kaplan-Meier survival curves were plotted to show the differences in of survival curves between the high and low expression group.

### Immunofluorescence staining

To confirm the endothelial and fibroblast subtypes in gastric tumors, we performed immunofluorescence staining of CTHRC1, ACKR1 and PRGFRB in five tumor samples. Serial sections (~4 μm) from formalin-fixed paraffin-embedded tumor tissues were stained using standard protocols. Anti-CD31 (mouse, 1:50, Proteintech, Ag1787, lot number: 66065-1-Ig) was used to stain endothelial cells. The following antibodies and dilutions were used to detect the corresponding proteins: anti-CTHRC1 (rabbit, 1:50, Proteintech, Ag9812, lot number: 16534-1-AP), anti-PDGFR B (mouse, 1:50, Abcam, ab51869, lot number: MM0014-5F66), and anti-ACKR1 (rabbit, 5 µg/ml, Abcam, ab58965). DAPI was used to stain cell nuclei.

### Replication analysis

To confirm our findings, we used three published scRNA-seq datasets (PMID 32532891, 34385296 and 34933901) to verify that the cells subpopulations identified in our datasets could represent the cellular heterozygosity of gastric cancer. For study of PMID 34933901, we only used pre-treatment samples in the replication analysis. The same cell clustering pipelines were used to re-analyze these three datasets. For T cells, we performed clustering for each dataset separately. For myeloid, fibroblast and endothelial cells, we aggregated cells from the three datasets into a single data matrix, then performed cell clustering.

## Supplementary Material

Supplementary figures.

Supplementary tables.
