# Supplementary material for: Single-cell landscape reveals active cell subtypes and their interaction in the tumor microenvironment of gastric cancer
Source: Theranostics. 2022 May 9;12(8):3818–33. doi: 10.7150/thno.71833 (PMC9131288; doi:10.7150/thno.71833)
Supplement: Supplementary file 1 — Supplementary figures. [file thnov12p3818s1.pdf]

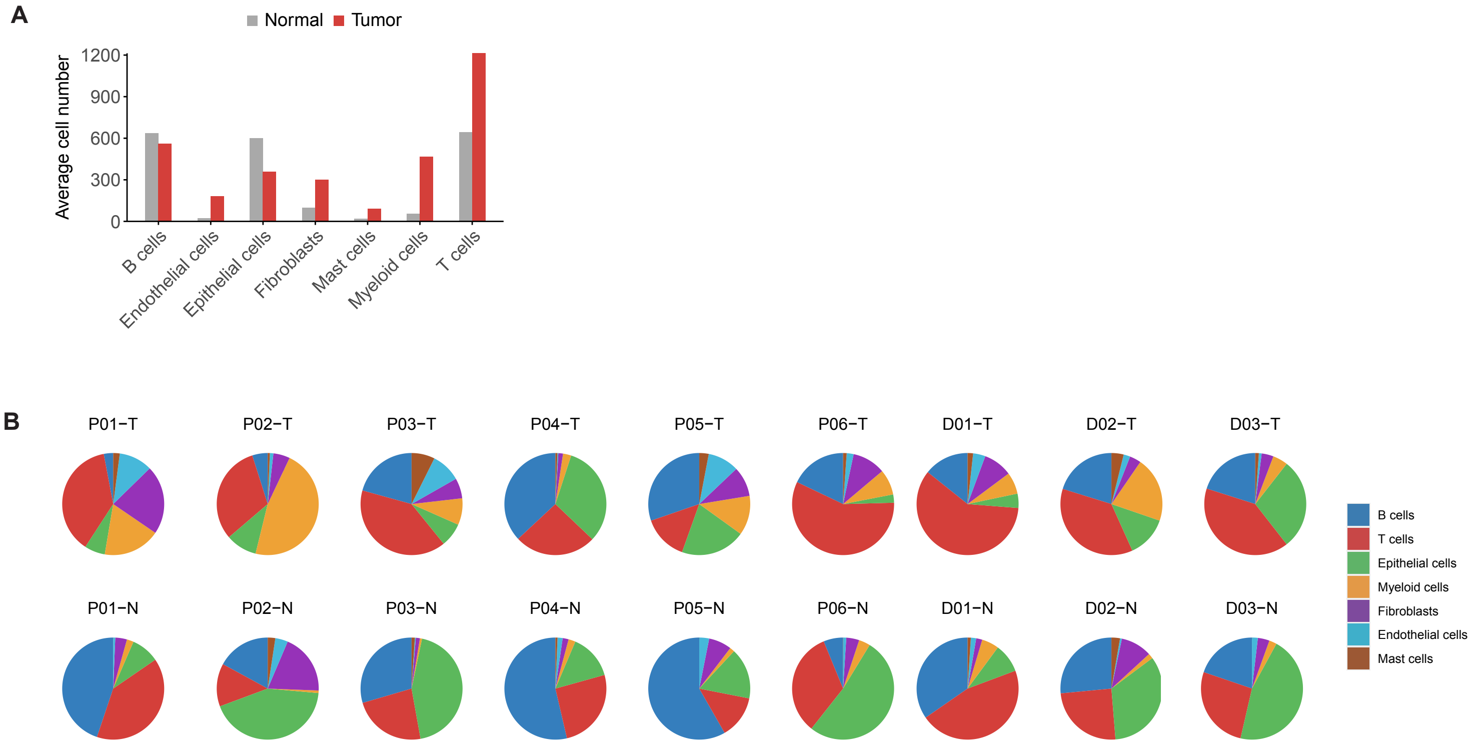
A
Normal
Tumor
Average cell number
1200
900
600
300
0
B cells
Endothelial cells
Epithelial cells
Fibroblasts
Mast cells
Myeloid cells
T cells
B
P01−T
P02−T
P03−T
P04−T
P05−T
P06−T
D01−T
D02−T
D03−T
P01−N
P02−N
P03−N
P04−N
P05−N
P06−N
D01−N
D02−N
D03−N
B cells
T cells
Epithelial cells
Myeloid cells
Fibroblasts
Endothelial cells
Mast cells

Figure S1

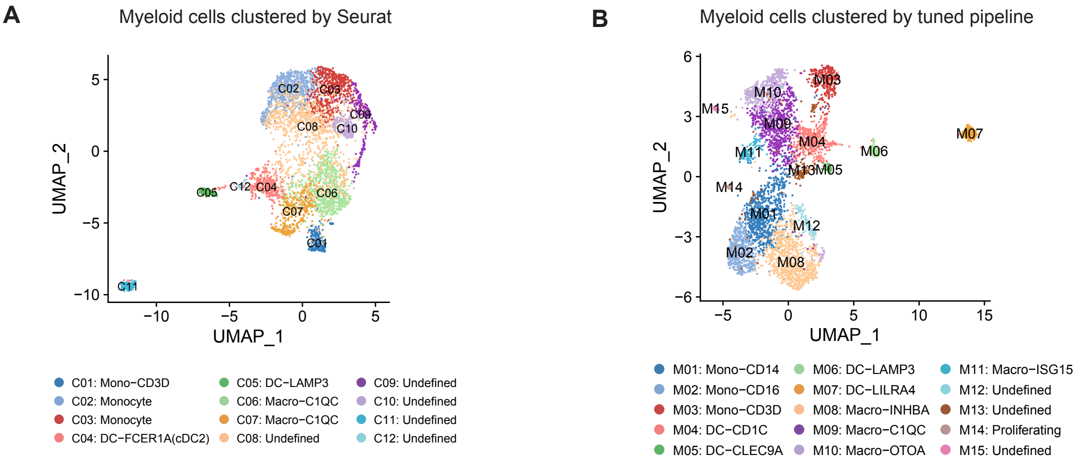
A
Myeloid cells clustered by Seurat
UMAP_1
UMAP_2
C01: Mono−CD3D
C02: Monocyte
C03: Monocyte
C04: DC−FCER1A(cDC2)
C05: DC−LAMP3
C06: Macro−C1QC
C07: Macro−C1QC
C08: Undefined
C09: Undefined
C10: Undefined
C11: Undefined
C12: Undefined
B
Myeloid cells clustered by tuned pipeline
UMAP_1
UMAP_2
M01: Mono−CD14
M02: Mono−CD16
M03: Mono−CD3D
M04: DC−CD1C
M05: DC−CLEC9A
M06: DC−LAMP3
M07: DC−LILRA4
M08: Macro−INHBA
M09: Macro−C1QC
M10: Macro−OTOA
M11: Macro−ISG15
M12: Undefined
M13: Undefined
M14: Proliferating
M15: Undefined

Figure S2

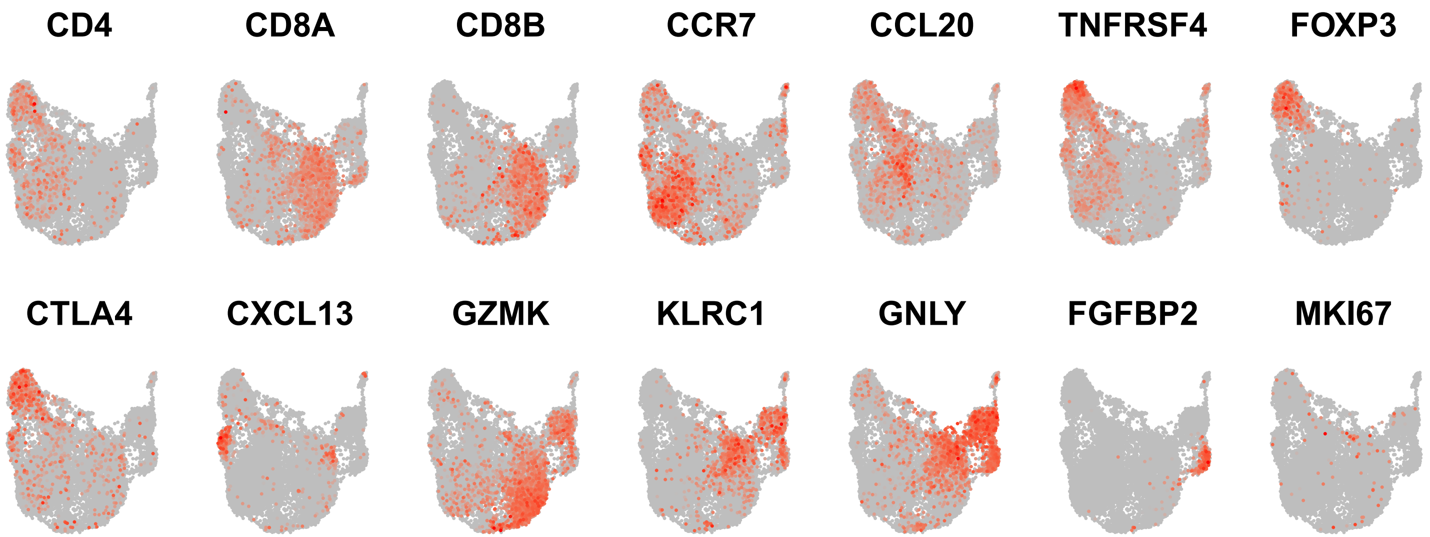
CD4
CD8A
CD8B
CCR7
CCL20
TNFRSF4
FOXP3
CTLA4
CXCL13
GZMK
KLRC1
GNLY
FGFBP2
MKI67

Figure S3

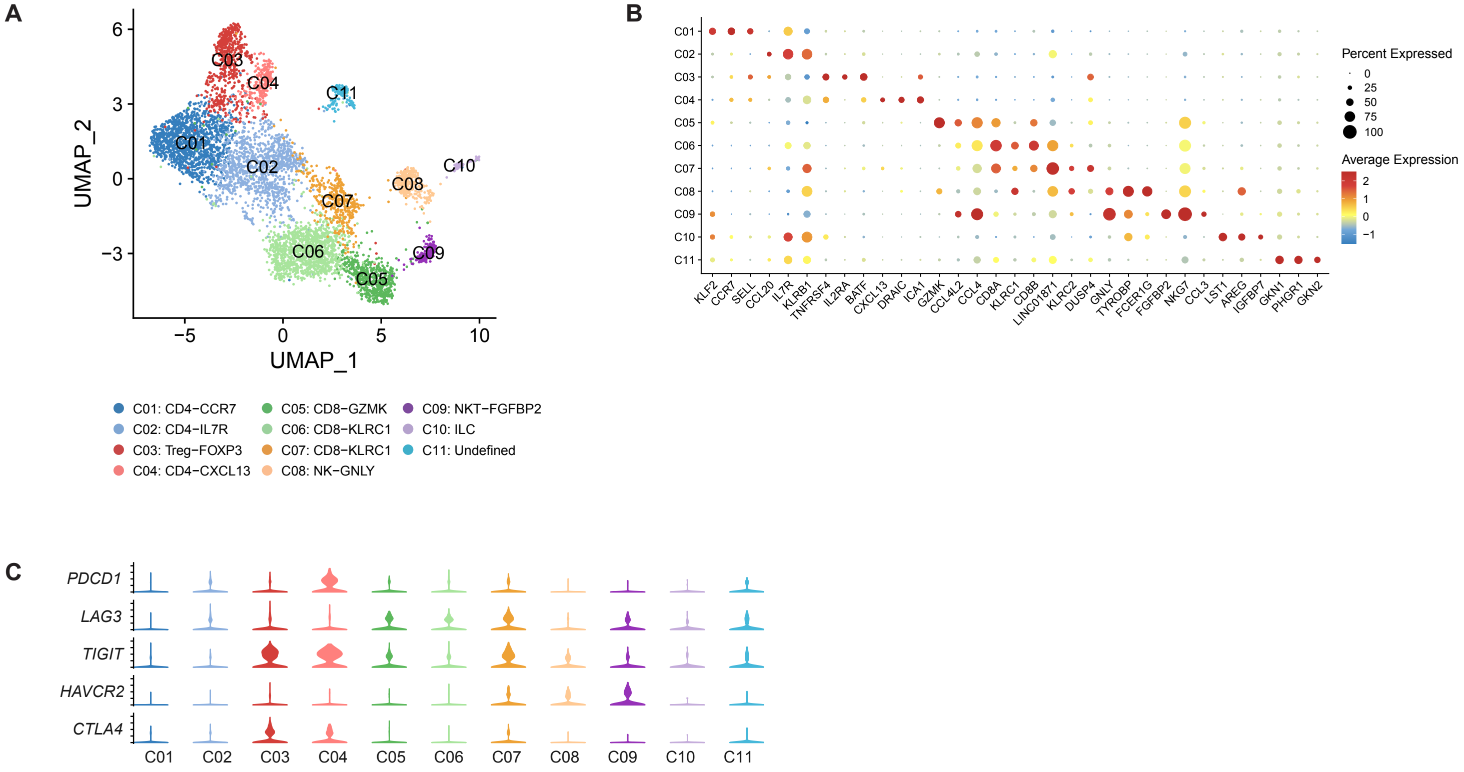
A
B
C
UMAP_1
UMAP_2
C01: CD4−CCR7
C02: CD4−IL7R
C03: Treg−FOXP3
C04: CD4−CXCL13
C05: CD8−GZMK
C06: CD8−KLRC1
C07: CD8−KLRC1
C08: NK−GNLY
C09: NKT−FGFBP2
C10: ILC
C11: Undefined
Percent Expressed
Average Expression
KLF2 CCR7 SELL CCL20 IL7R KLRB1 TNFRSF4 IL2RA BATF CXCL13 DRAIC ICA1 GZMK CCL4L2 CCL4 CD8A KLRC1 CD8B LINC01871 KLRC2 DUSP4 GNLY TYROBP FCER1G FGFBP2 NKG7 CCL3 LST1 AREG IGFBP7 GKN1 PHGR1 GKN2
PDCD1
LAG3
TIGIT
HAVCR2
CTLA4

Figure S4

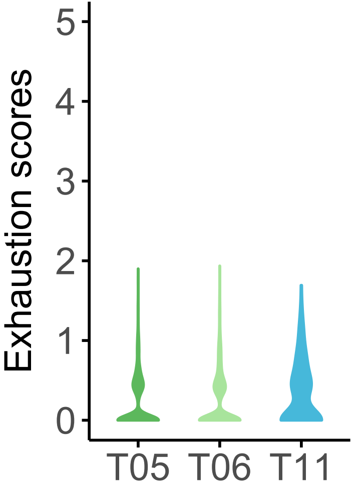

Figure S5

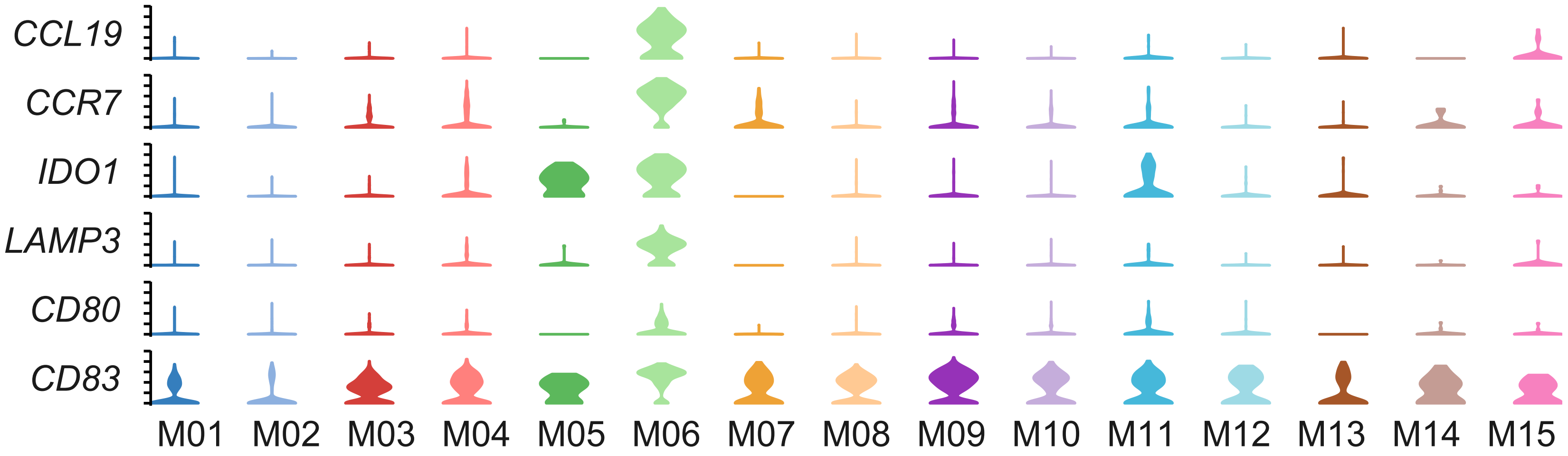
CCL19
CCR7
IDO1
LAMP3
CD80
CD83
M01
M02
M03
M04
M05
M06
M07
M08
M09
M10
M11
M12
M13
M14
M15

Figure S6

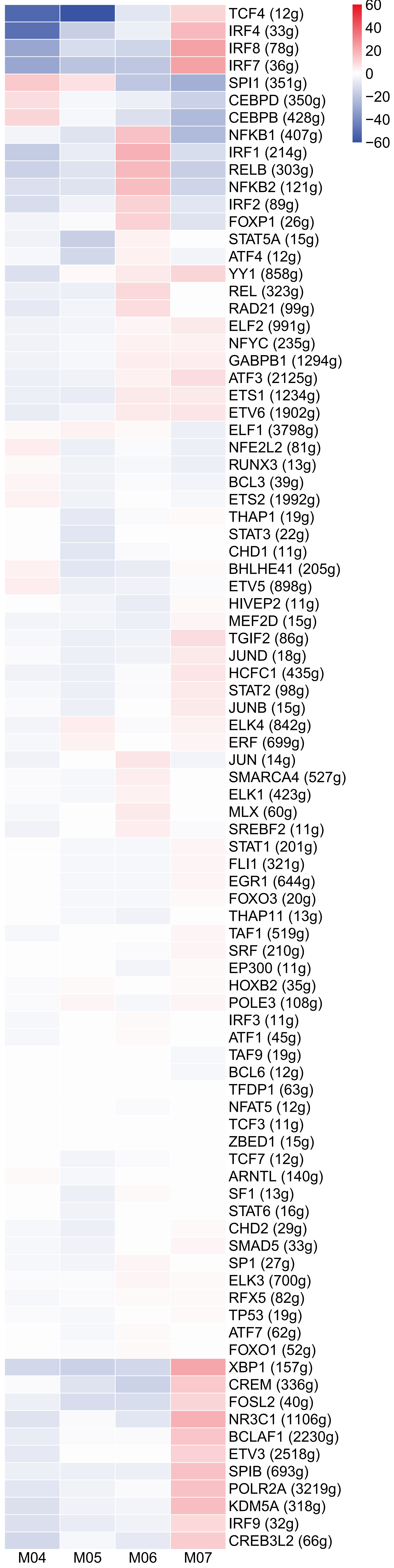
TCF4 (12g)
IRF4 (33g)
IRF8 (78g)
IRF7 (36g)
SPI1 (351g)
CEBPD (350g)
CEBPB (428g)
NFKB1 (407g)
IRF1 (214g)
RELB (303g)
NFKB2 (121g)
IRF2 (89g)
FOXP1 (26g)
STAT5A (15g)
ATF4 (12g)
YY1 (858g)
REL (323g)
RAD21 (99g)
ELF2 (991g)
NFYC (235g)
GABPB1 (1294g)
ATF3 (2125g)
ETS1 (1234g)
ETV6 (1902g)
ELF1 (3798g)
NFE2L2 (81g)
RUNX3 (13g)
BCL3 (39g)
ETS2 (1992g)
THAP1 (19g)
STAT3 (22g)
CHD1 (11g)
BHLHE41 (205g)
ETV5 (898g)
HIVEP2 (11g)
MEF2D (15g)
TGIF2 (86g)
JUND (18g)
HCFC1 (435g)
STAT2 (98g)
JUNB (15g)
ELK4 (842g)
ERF (699g)
JUN (14g)
SMARCA4 (527g)
ELK1 (423g)
MLX (60g)
SREBF2 (11g)
STAT1 (201g)
FLI1 (321g)
EGR1 (644g)
FOXO3 (20g)
THAP11 (13g)
TAF1 (519g)
SRF (210g)
EP300 (11g)
HOXB2 (35g)
POLE3 (108g)
IRF3 (11g)
ATF1 (45g)
TAF9 (19g)
BCL6 (12g)
TFDP1 (63g)
NFAT5 (12g)
TCF3 (11g)
ZBED1 (15g)
TCF7 (12g)
ARNTL (140g)
SF1 (13g)
STAT6 (16g)
CHD2 (29g)
SMAD5 (33g)
SP1 (27g)
ELK3 (700g)
RFX5 (82g)
TP53 (19g)
ATF7 (62g)
FOXO1 (52g)
XBP1 (157g)
CREM (336g)
FOSL2 (40g)
NR3C1 (1106g)
BCLAF1 (2230g)
ETV3 (2518g)
SPIB (693g)
POLR2A (3219g)
KDM5A (318g)
IRF9 (32g)
CREB3L2 (66g)
M04
M05
M06
M07
60
40
20
0
−20
−40
−60

Figure S7

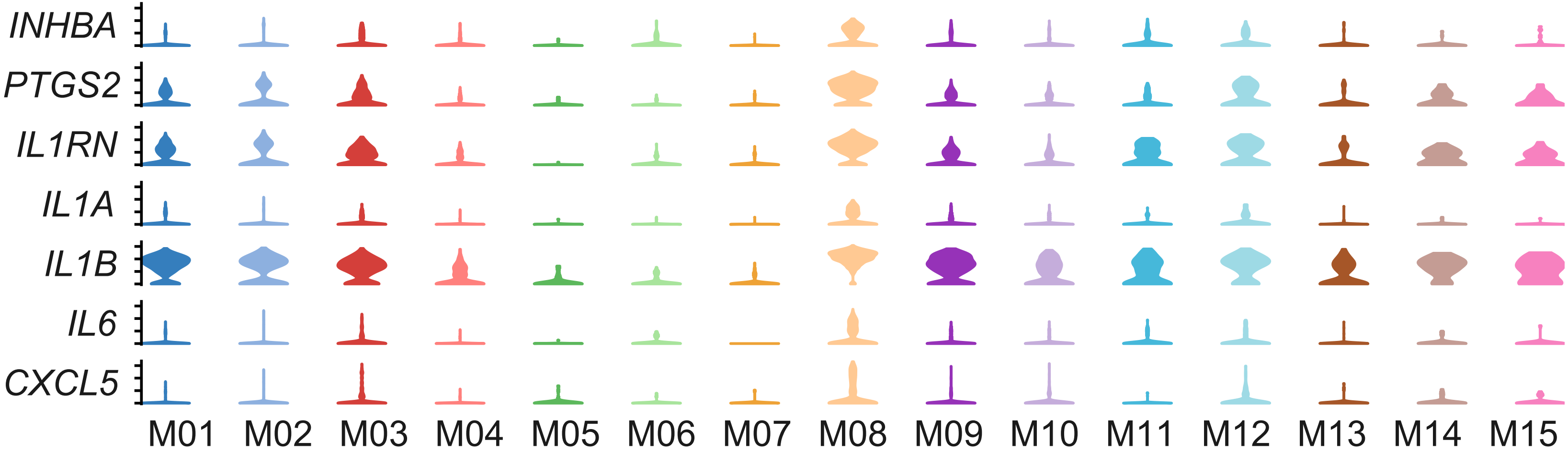
INHBA
PTGS2
IL1RN
IL1A
IL1B
IL6
CXCL5
M01
M02
M03
M04
M05
M06
M07
M08
M09
M10
M11
M12
M13
M14
M15

Figure S8

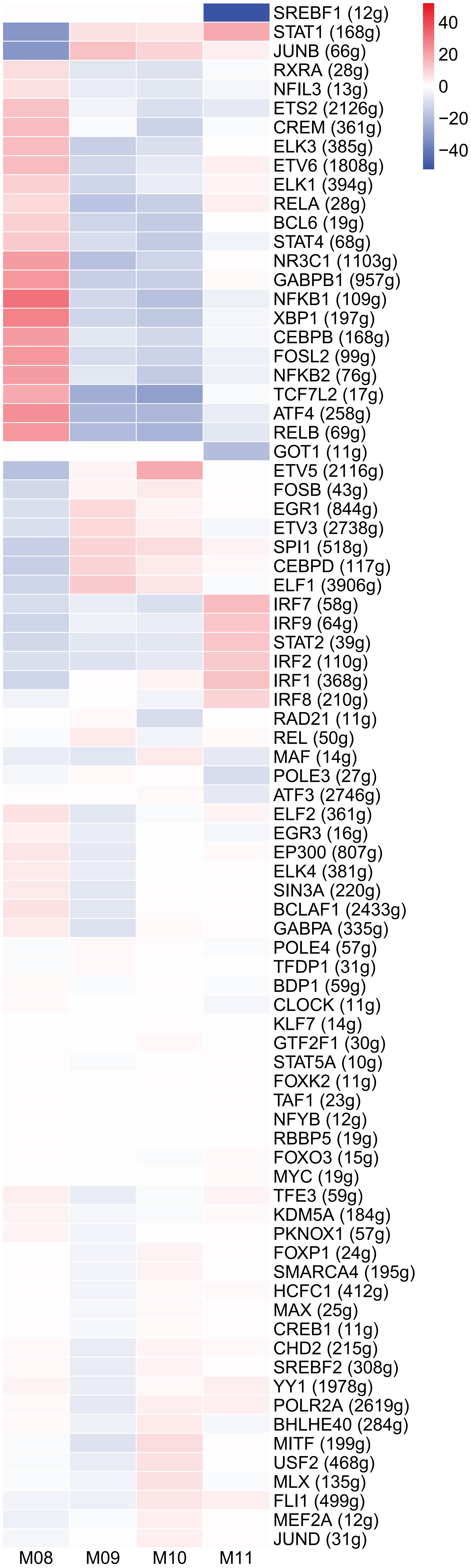
SREBF1 (12g)
STAT1 (168g)
JUNB (66g)
RXRA (28g)
NFIL3 (13g)
ETS2 (2126g)
CREM (361g)
ELK3 (385g)
ETV6 (1808g)
ELK1 (394g)
RELA (28g)
BCL6 (19g)
STAT4 (68g)
NR3C1 (1103g)
GABPB1 (957g)
NFKB1 (109g)
XBP1 (197g)
CEBPB (168g)
FOSL2 (99g)
NFKB2 (76g)
TCF7L2 (17g)
ATF4 (258g)
RELB (69g)
GOT1 (11g)
ETV5 (2116g)
FOSB (43g)
EGR1 (844g)
ETV3 (2738g)
SPI1 (518g)
CEBPD (117g)
ELF1 (3906g)
IRF7 (58g)
IRF9 (64g)
STAT2 (39g)
IRF2 (110g)
IRF1 (368g)
IRF8 (210g)
RAD21 (11g)
REL (50g)
MAF (14g)
POLE3 (27g)
ATF3 (2746g)
ELF2 (361g)
EGR3 (16g)
EP300 (807g)
ELK4 (381g)
SIN3A (220g)
BCLAF1 (2433g)
GABPA (335g)
POLE4 (57g)
TFDP1 (31g)
BDP1 (59g)
CLOCK (11g)
KLF7 (14g)
GTF2F1 (30g)
STAT5A (10g)
FOXK2 (11g)
TAF1 (23g)
NFYB (12g)
RBBP5 (19g)
FOXO3 (15g)
MYC (19g)
TFE3 (59g)
KDM5A (184g)
PKNOX1 (57g)
FOXP1 (24g)
SMARCA4 (195g)
HCFC1 (412g)
MAX (25g)
CREB1 (11g)
CHD2 (215g)
SREBF2 (308g)
YY1 (1978g)
POLR2A (2619g)
BHLHE40 (284g)
MITF (199g)
USF2 (468g)
MLX (135g)
FLI1 (499g)
MEF2A (12g)
JUND (31g)
M08
M09
M10
M11
40
20
0
−20
−40

Figure S9

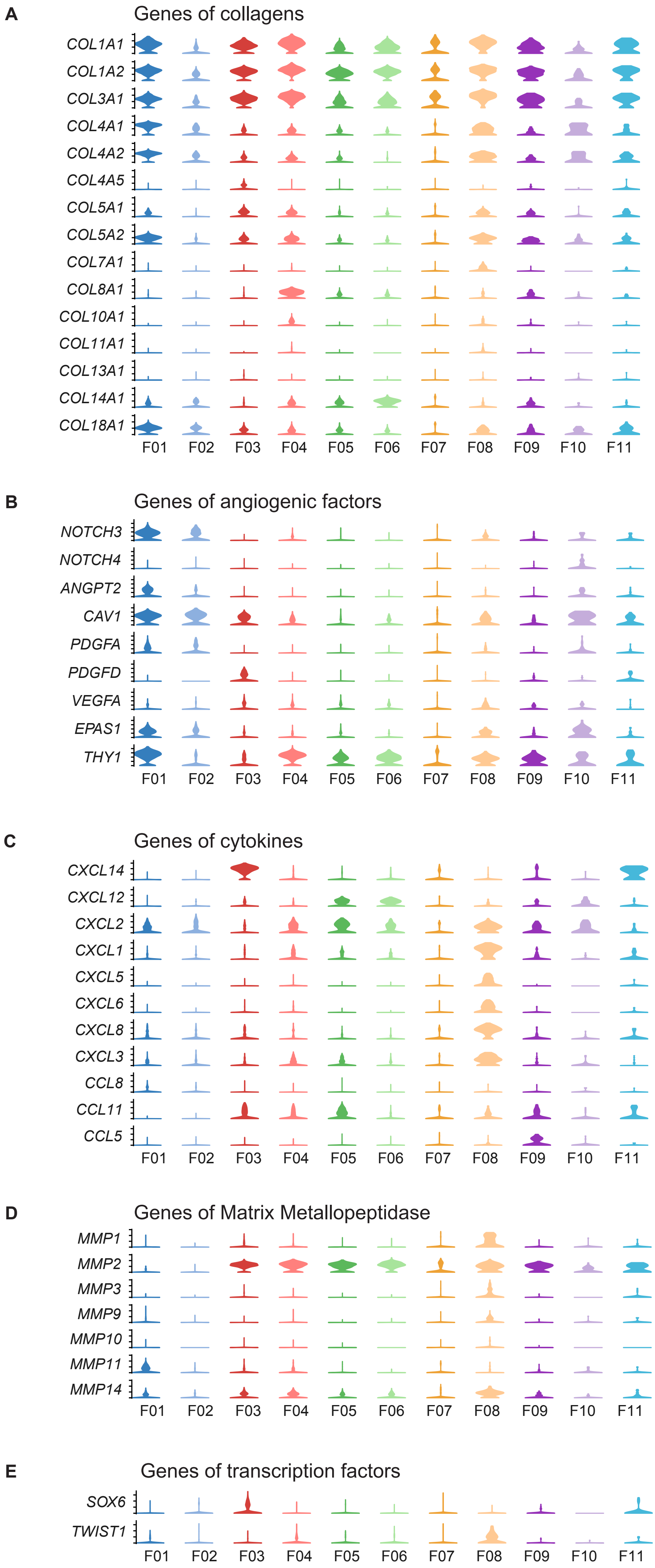
A
Genes of collagens
COL1A1
COL1A2
COL3A1
COL4A1
COL4A2
COL4A5
COL5A1
COL5A2
COL7A1
COL8A1
COL10A1
COL11A1
COL13A1
COL14A1
COL18A1
F01 F02 F03 F04 F05 F06 F07 F08 F09 F10 F11
B
Genes of angiogenic factors
NOTCH3
NOTCH4
ANGPT2
CAV1
PDGFA
PDGFD
VEGFA
EPAS1
THY1
F01 F02 F03 F04 F05 F06 F07 F08 F09 F10 F11
C
Genes of cytokines
CXCL14
CXCL12
CXCL2
CXCL1
CXCL5
CXCL6
CXCL8
CXCL3
CCL8
CCL11
CCL5
F01 F02 F03 F04 F05 F06 F07 F08 F09 F10 F11
D
Genes of Matrix Metallopeptidase
MMP1
MMP2
MMP3
MMP9
MMP10
MMP11
MMP14
F01 F02 F03 F04 F05 F06 F07 F08 F09 F10 F11
E
Genes of transcription factors
SOX6
TWIST1
F01 F02 F03 F04 F05 F06 F07 F08 F09 F10 F11

Figure S10

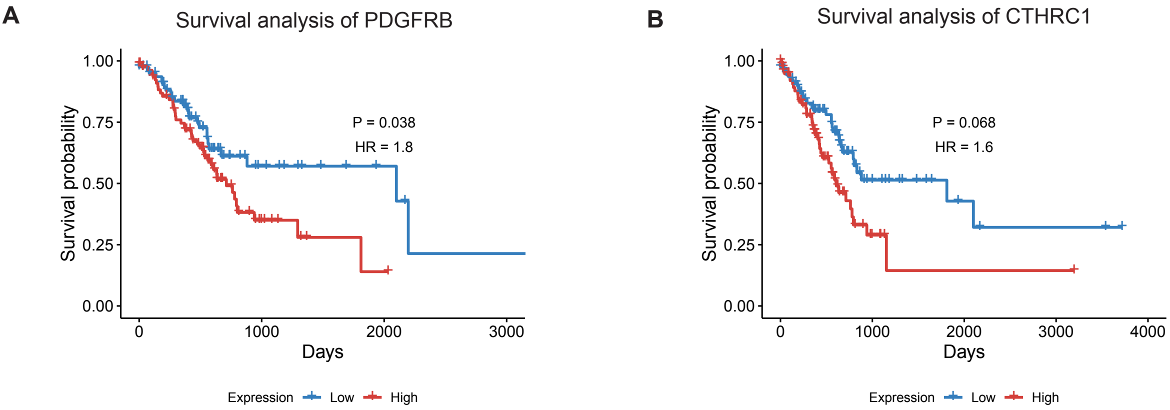
A
Survival analysis of PDGFRB
1.00
0.75
0.50
0.25
0.00
Survival probability
P = 0.038
HR = 1.8
0
1000
2000
3000
Days
Expression
Low
High
B
Survival analysis of CTHRC1
1.00
0.75
0.50
0.25
0.00
Survival probability
P = 0.068
HR = 1.6
0
1000
2000
3000
4000
Days
Expression
Low
High

Figure S11

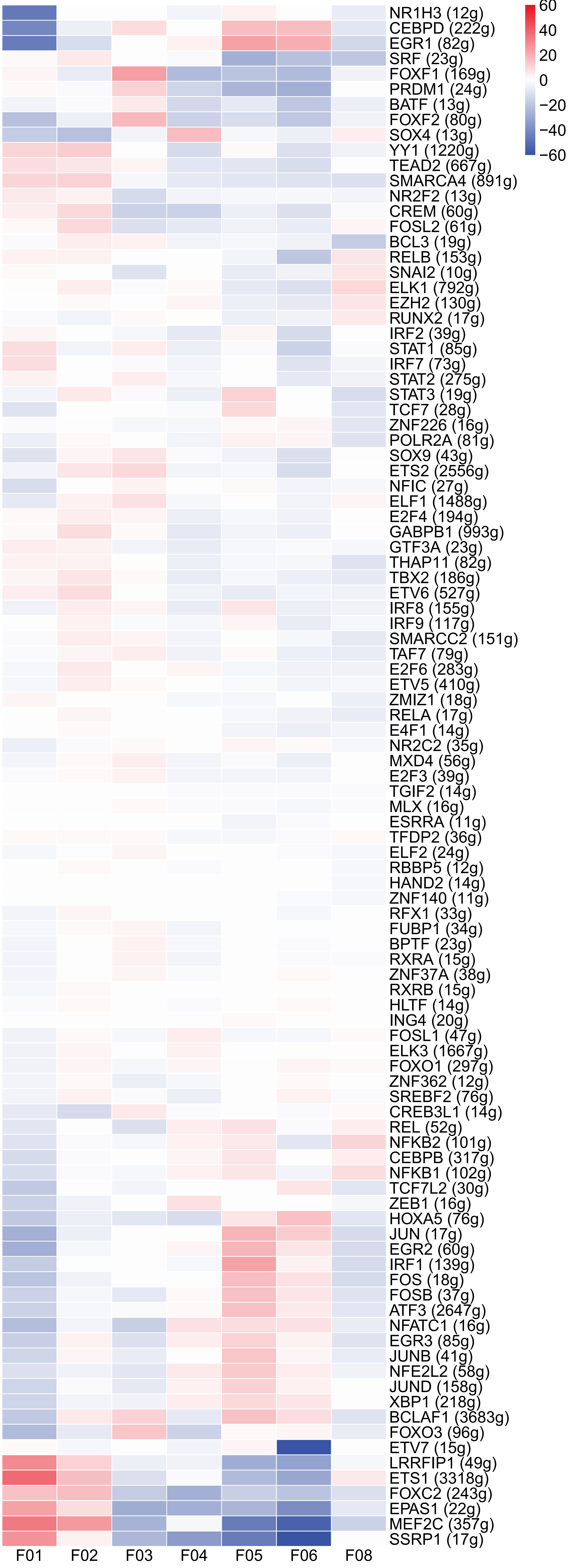
60
40
20
0
−20
−40
−60
NR1H3 (12g)
CEBPD (222g)
EGR1 (82g)
SRF (23g)
FOXF1 (169g)
PRDM1 (24g)
BATF (13g)
FOXF2 (80g)
SOX4 (13g)
YY1 (1220g)
TEAD2 (667g)
SMARCA4 (891g)
NR2F2 (13g)
CREM (60g)
FOSL2 (61g)
BCL3 (19g)
RELB (153g)
SNAI2 (10g)
ELK1 (792g)
EZH2 (130g)
RUNX2 (17g)
IRF2 (39g)
STAT1 (85g)
IRF7 (73g)
STAT2 (275g)
STAT3 (19g)
TCF7 (28g)
ZNF226 (16g)
POLR2A (81g)
SOX9 (43g)
ETS2 (2556g)
NFIC (27g)
ELF1 (1488g)
E2F4 (194g)
GABPB1 (993g)
GTF3A (23g)
THAP11 (82g)
TBX2 (186g)
ETV6 (527g)
IRF8 (155g)
IRF9 (117g)
SMARCC2 (151g)
TAF7 (79g)
E2F6 (283g)
ETV5 (410g)
ZMIZ1 (18g)
RELA (17g)
E4F1 (14g)
NR2C2 (35g)
MXD4 (56g)
E2F3 (39g)
TGIF2 (14g)
MLX (16g)
ESRRA (11g)
TFDP2 (36g)
ELF2 (24g)
RBBP5 (12g)
HAND2 (14g)
ZNF140 (11g)
RFX1 (33g)
FUBP1 (34g)
BPTF (23g)
RXRA (15g)
ZNF37A (38g)
RXRB (15g)
HLTF (14g)
ING4 (20g)
FOSL1 (47g)
ELK3 (1667g)
FOXO1 (297g)
ZNF362 (12g)
SREBF2 (76g)
CREB3L1 (14g)
REL (52g)
NFKB2 (101g)
CEBPB (317g)
NFKB1 (102g)
TCF7L2 (30g)
ZEB1 (16g)
HOXA5 (76g)
JUN (17g)
EGR2 (60g)
IRF1 (139g)
FOS (18g)
FOSB (37g)
ATF3 (2647g)
NFATC1 (16g)
EGR3 (85g)
JUNB (41g)
NFE2L2 (58g)
JUND (158g)
XBP1 (218g)
BCLAF1 (3683g)
FOXO3 (96g)
ETV7 (15g)
LRRFIP1 (49g)
ETS1 (3318g)
FOXC2 (243g)
EPAS1 (22g)
MEF2C (357g)
SSRP1 (17g)
F01
F02
F03
F04
F05
F06
F08

Figure S12

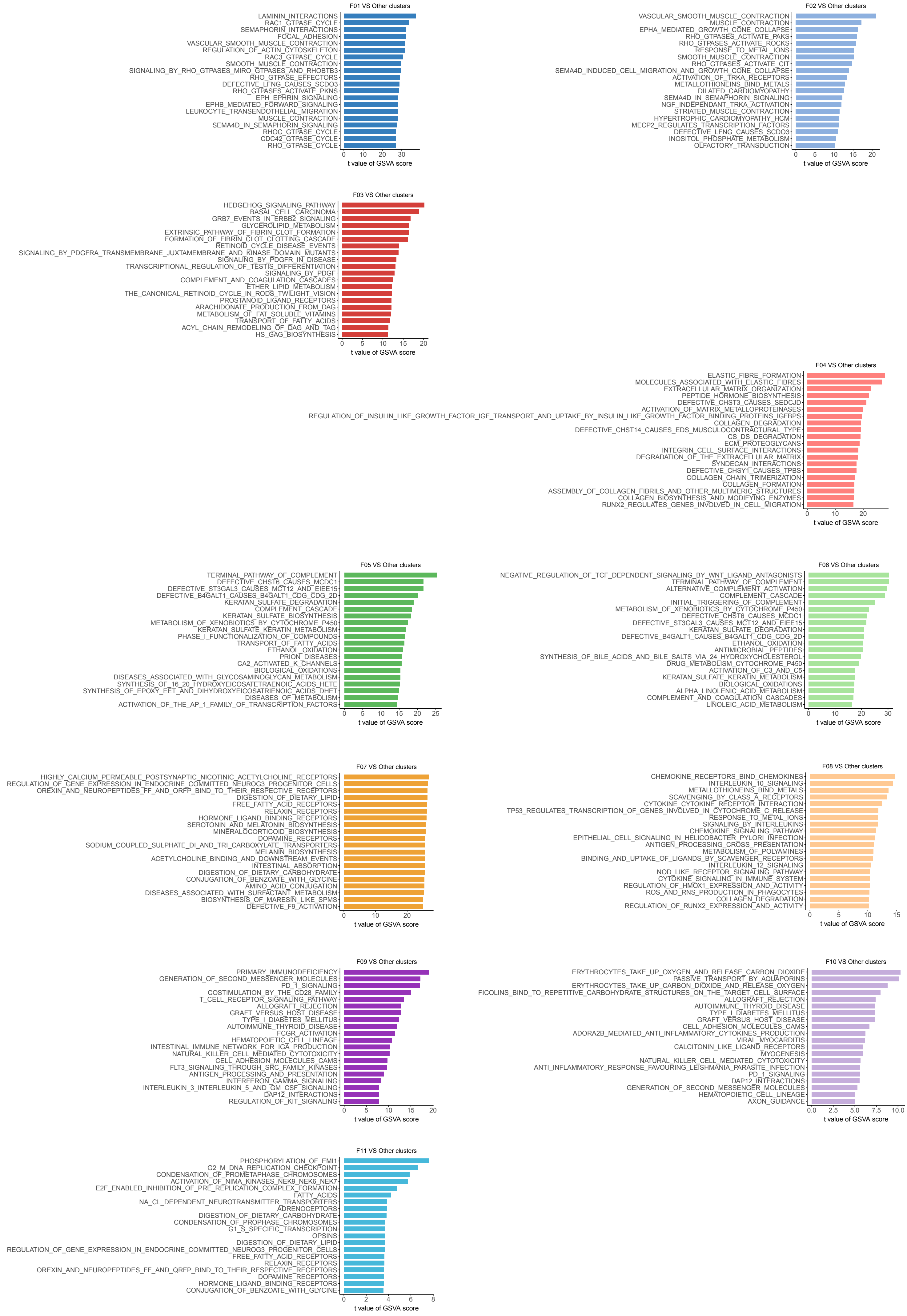
F01 VS Other clusters
LAMININ_INTERACTIONS
RAC1_GTPASE_CYCLE
SEMAPHORIN_INTERACTIONS
FOCAL_ADHESION
VASCULAR_SMOOTH_MUSCLE_CONTRACTION
REGULATION_OF_ACTIN_CYTOSKELETON
RAC3_GTPASE_CYCLE
SMOOTH_MUSCLE_CONTRACTION
SIGNALING_BY_RHO_GTPASES_MIRO_GTPASES_AND_RHOBTB3
RHO_GTPASE_EFFECTORS
DEFECTIVE_LFNG_CAUSES_SCDO3
RHO_GTPASES_ACTIVATE_PKNS
EPH_EPHRIN_SIGNALING
EPHB_MEDIATED_FORWARD_SIGNALING
LEUKOCYTE_TRANSENDOTHELIAL_MIGRATION
MUSCLE_CONTRACTION
SEMA4D_IN_SEMAPHORIN_SIGNALING
RHOC_GTPASE_CYCLE
CDC42_GTPASE_CYCLE
RHOB_GTPASE_CYCLE
0 10 20 30
t value of GSVA score
F02 VS Other clusters
VASCULAR_SMOOTH_MUSCLE_CONTRACTION
MUSCLE_CONTRACTION
EPHA_MEDIATED_GROWTH_CONE_COLLAPSE
RHO_GTPASES_ACTIVATE_PAKS
RHO_GTPASES_ACTIVATE_ROCKS
RESPONSE_TO_METAL_IONS
SMOOTH_MUSCLE_CONTRACTION
RHO_GTPASES_ACTIVATE_CIT
SEMA4D_INDUCED_CELL_MIGRATION_AND_GROWTH_CONE_COLLAPSE
ACTIVATION_OF_TRKA_RECEPTORS
METALLOTHIONEINS_BIND_METALS
DILATED_CARDIOMYOPATHY
SEMA4D_IN_SEMAPHORIN_SIGNALING
NGF_INDEPENDANT_TRKA_ACTIVATION
STRIATED_MUSCLE_CONTRACTION
HYPERTROPHIC_CARDIOMYOPATHY_HCM
MECP2_REGULATES_TRANSCRIPTION_FACTORS
DEFECTIVE_LFNG_CAUSES_SCDO3
INOSITOL_PHOSPHATE_METABOLISM
OLFACTORY_TRANSDUCTION
0 5 10 15 20
t value of GSVA score
F03 VS Other clusters
HEDGEHOG_SIGNALING_PATHWAY
BASAL_CELL_CARCINOMA
GRB7_EVENTS_IN_ERBB2_SIGNALING
GLYCEROLIPID_METABOLISM
EXTRINSIC_PATHWAY_OF_FIBRIN_CLOT_FORMATION
FORMATION_OF_FIBRIN_CLOT_CLOTTING_CASCADE
RETINOID_CYCLE_DISEASE_EVENTS
SIGNALING_BY_PDGFRA_TRANSMEMBRANE_JUXTAMEMBRANE_AND_KINASE_DOMAIN_MUTANTS
SIGNALING_BY_PDGFR_IN_DISEASE
TRANSCRIPTIONAL_REGULATION_OF_TESTIS_DIFFERENTIATION
SIGNALING_BY_PDGF
COMPLEMENT_AND_COAGULATION_CASCADES
ETHER_LIPID_METABOLISM
THE_CANONICAL_RETINOID_CYCLE_IN_RODS_TWILIGHT_VISION
PROSTANOID_LIGAND_RECEPTORS
ARACHIDONATE_PRODUCTION_FROM_DAG
METABOLISM_OF_FAT_SOLUBLE_VITAMINS
TRANSPORT_OF_FATTY_ACIDS
ACYL_CHAIN_REMODELING_OF_DAG_AND_TAG
HS_GAG_BIOSYNTHESIS
0 5 10 15 20
t value of GSVA score
F04 VS Other clusters
ELASTIC_FIBRE_FORMATION
MOLECULES_ASSOCIATED_WITH_ELASTIC_FIBRES
EXTRACELLULAR_MATRIX_ORGANIZATION
PEPTIDE_HORMONE_BIOSYNTHESIS
DEFECTIVE_CHST3_CAUSES_SEDCJD
ACTIVATION_OF_MATRIX_METALLOPROTEINASES
REGULATION_OF_INSULIN_LIKE_GROWTH_FACTOR_IGF_TRANSPORT_AND_UPTAKE_BY_INSULIN_LIKE_GROWTH_FACTOR_BINDING_PROTEINS_IGFBPS
COLLAGEN_DEGRADATION
DEFECTIVE_CHST14_CAUSES_EDS_MUSCULOCONTRACTURAL_TYPE
CS_DS_DEGRADATION
ECM_PROTEOGLYCANS
INTEGRIN_CELL_SURFACE_INTERACTIONS
DEGRADATION_OF_THE_EXTRACELLULAR_MATRIX
SYNDECAN_INTERACTIONS
DEFECTIVE_CHSY1_CAUSES_TPBS
COLLAGEN_CHAIN_TRIMERIZATION
COLLAGEN_FORMATION
ASSEMBLY_OF_COLLAGEN_FIBRILS_AND_OTHER_MULTIMERIC_STRUCTURES
COLLAGEN_BIOSYNTHESIS_AND_MODIFYING_ENZYMES
RUNX2_REGULATES_GENES_INVOLVED_IN_CELL_MIGRATION
0 10 20
t value of GSVA score
F05 VS Other clusters
TERMINAL_PATHWAY_OF_COMPLEMENT
DEFECTIVE_CHST6_CAUSES_MCDC1
DEFECTIVE_ST3GAL3_CAUSES_MCT12_AND_EIEE15
DEFECTIVE_B4GALT1_CAUSES_B4GALT1_CDG_CDG_2D
KERATAN_SULFATE_DEGRADATION
COMPLEMENT_CASCADE
KERATAN_SULFATE_BIOSYNTHESIS
METABOLISM_OF_XENOBIOTICS_BY_CYTOCHROME_P450
KERATAN_SULFATE_KERATIN_METABOLISM
PHASE_I_FUNCTIONALIZATION_OF_COMPOUNDS
TRANSPORT_OF_FATTY_ACIDS
ETHANOL_OXIDATION
PRION_DISEASES
CA2_ACTIVATED_K_CHANNELS
BIOLOGICAL_OXIDATIONS
DISEASES_ASSOCIATED_WITH_GLYCOSAMINOGLYCAN_METABOLISM
SYNTHESIS_OF_16_20_HYDROXYEICOSATETRAENOIC_ACIDS_HETE
SYNTHESIS_OF_EPOXY_EET_AND_DIHYDROXYEICOSATRIENOIC_ACIDS_DHET
DISEASES_OF_METABOLISM
ACTIVATION_OF_THE_AP_1_FAMILY_OF_TRANSCRIPTION_FACTORS
0 5 10 15 20 25
t value of GSVA score
F06 VS Other clusters
NEGATIVE_REGULATION_OF_TCF_DEPENDENT_SIGNALING_BY_WNT_LIGAND_ANTAGONISTS
TERMINAL_PATHWAY_OF_COMPLEMENT
ALTERNATIVE_COMPLEMENT_ACTIVATION
COMPLEMENT_CASCADE
INITIAL_TRIGGERING_OF_COMPLEMENT
METABOLISM_OF_XENOBIOTICS_BY_CYTOCHROME_P450
DEFECTIVE_CHST6_CAUSES_MCDC1
DEFECTIVE_ST3GAL3_CAUSES_MCT12_AND_EIEE15
KERATAN_SULFATE_DEGRADATION
DEFECTIVE_B4GALT1_CAUSES_B4GALT1_CDG_CDG_2D
ETHANOL_OXIDATION
ANTIMICROBIAL_PEPTIDES
SYNTHESIS_OF_BILE_ACIDS_AND_BILE_SALTS_VIA_24_HYDROXYCHOLESTEROL
DRUG_METABOLISM_CYTOCHROME_P450
ACTIVATION_OF_C3_AND_C5
KERATAN_SULFATE_KERATIN_METABOLISM
BIOLOGICAL_OXIDATIONS
ALPHA_LINOLENIC_ACID_METABOLISM
COMPLEMENT_AND_COAGULATION_CASCADES
LINOLEIC_ACID_METABOLISM
0 10 20 30
t value of GSVA score
F07 VS Other clusters
HIGHLY_CALCIUM_PERMEABLE_POSTSYNAPTIC_NICOTINIC_ACETYLCHOLINE_RECEPTORS
REGULATION_OF_GENE_EXPRESSION_IN_ENDOCRINE_COMMITTED_NEUROG3_PROGENITOR_CELLS
OREXIN_AND_NEUROPEPTIDES_FF_AND_QRFP_BIND_TO_THEIR_RESPECTIVE_RECEPTORS
DIGESTION_OF_DIETARY_LIPID
FREE_FATTY_ACID_RECEPTORS
RELAXIN_RECEPTORS
HORMONE_LIGAND_BINDING_RECEPTORS
SEROTONIN_AND_MELATONIN_BIOSYNTHESIS
MINERALOCORTICOID_BIOSYNTHESIS
DOPAMINE_RECEPTORS
SODIUM_COUPLED_SULPHATE_DI_AND_TRI_CARBOXYLATE_TRANSPORTERS
MELANIN_BIOSYNTHESIS
ACETYLCHOLINE_BINDING_AND_DOWNSTREAM_EVENTS
INTESTINAL_ABSORPTION
DIGESTION_OF_DIETARY_CARBOHYDRATE
CONJUGATION_OF_BENZOATE_WITH_GLYCINE
AMINO_ACID_CONJUGATION
DISEASES_ASSOCIATED_WITH_SURFACTANT_METABOLISM
BIOSYNTHESIS_OF_MARESIN_LIKE_SPMS
DEFECTIVE_F9_ACTIVATION
0 10 20
t value of GSVA score
F08 VS Other clusters
CHEMOKINE_RECEPTORS_BIND_CHEMOKINES
INTERLEUKIN_10_SIGNALING
METALLOTHIONEINS_BIND_METALS
SCAVENGING_BY_CLASS_A_RECEPTORS
CYTOKINE_CYTOKINE_RECEPTOR_INTERACTION
TP53_REGULATES_TRANSCRIPTION_OF_GENES_INVOLVED_IN_CYTOCHROME_C_RELEASE
RESPONSE_TO_METAL_IONS
SIGNALING_BY_INTERLEUKINS
CHEMOKINE_SIGNALING_PATHWAY
EPITHELIAL_CELL_SIGNALING_IN_HELICOBACTER_PYLORI_INFECTION
ANTIGEN_PROCESSING_CROSS_PRESENTATION
METABOLISM_OF_POLYAMINES
BINDING_AND_UPTAKE_OF_LIGANDS_BY_SCAVENGER_RECEPTORS
INTERLEUKIN_12_SIGNALING
NOD_LIKE_RECEPTOR_SIGNALING_PATHWAY
CYTOKINE_SIGNALING_IN_IMMUNE_SYSTEM
REGULATION_OF_HMOX1_EXPRESSION_AND_ACTIVITY
ROS_AND_RNS_PRODUCTION_IN_PHAGOCYTES
COLLAGEN_DEGRADATION
REGULATION_OF_RUNX2_EXPRESSION_AND_ACTIVITY
0 5 10 15
t value of GSVA score
F09 VS Other clusters
PRIMARY_IMMUNODEFICIENCY
GENERATION_OF_SECOND_MESSENGER_MOLECULES
PD_1_SIGNALING
COSTIMULATION_BY_THE_CD28_FAMILY
T_CELL_RECEPTOR_SIGNALING_PATHWAY
ALLOGRAFT_REJECTION
GRAFT_VERSUS_HOST_DISEASE
TYPE_I_DIABETES_MELLITUS
AUTOIMMUNE_THYROID_DISEASE
FCGR_ACTIVATION
HEMATOPOIETIC_CELL_LINEAGE
INTESTINAL_IMMUNE_NETWORK_FOR_IGA_PRODUCTION
NATURAL_KILLER_CELL_MEDIATED_CYTOTOXICITY
CELL_ADHESION_MOLECULES_CAMS
FLT3_SIGNALING_THROUGH_SRC_FAMILY_KINASES
ANTIGEN_PROCESSING_AND_PRESENTATION
INTERFERON_GAMMA_SIGNALING
INTERLEUKIN_3_INTERLEUKIN_5_AND_GM_CSF_SIGNALING
DAP12_INTERACTIONS
REGULATION_OF_KIT_SIGNALING
0 5 10 15 20
t value of GSVA score
F10 VS Other clusters
ERYTHROCYTES_TAKE_UP_OXYGEN_AND_RELEASE_CARBON_DIOXIDE
PASSIVE_TRANSPORT_BY_AQUAPORINS
ERYTHROCYTES_TAKE_UP_CARBON_DIOXIDE_AND_RELEASE_OXYGEN
FICOLINS_BIND_TO_REPETITIVE_CARBOHYDRATE_STRUCTURES_ON_THE_TARGET_CELL_SURFACE
ALLOGRAFT_REJECTION
AUTOIMMUNE_THYROID_DISEASE
TYPE_I_DIABETES_MELLITUS
GRAFT_VERSUS_HOST_DISEASE
CELL_ADHESION_MOLECULES_CAMS
ADORA2B_MEDIATED_ANTI_INFLAMMATORY_CYTOKINES_PRODUCTION
VIRAL_MYOCARDITIS
CALCITONIN_LIKE_LIGAND_RECEPTORS
MYOGENESIS
NATURAL_KILLER_CELL_MEDIATED_CYTOTOXICITY
ANTI_INFLAMMATORY_RESPONSE_FAVOURING_LEISHMANIA_PARASITE_INFECTION
PD_1_SIGNALING
DAP12_INTERACTIONS
GENERATION_OF_SECOND_MESSENGER_MOLECULES
HEMATOPOIETIC_CELL_LINEAGE
AXON_GUIDANCE
0.0 2.5 5.0 7.5 10.0
t value of GSVA score
F11 VS Other clusters
PHOSPHORYLATION_OF_EMI1
G2_M_DNA_REPLICATION_CHECKPOINT
CONDENSATION_OF_PROMETAPHASE_CHROMOSOMES
ACTIVATION_OF_NIMA_KINASES_NEK9_NEK6_NEK7
E2F_ENABLED_INHIBITION_OF_PRE_REPLICATION_COMPLEX_FORMATION
FATTY_ACIDS
NA_CL_DEPENDENT_NEUROTRANSMITTER_TRANSPORTERS
ADRENOCEPTORS
DIGESTION_OF_DIETARY_CARBOHYDRATE
CONDENSATION_OF_PROPHASE_CHROMOSOMES
G1_S_SPECIFIC_TRANSCRIPTION
OPSINS
DIGESTION_OF_DIETARY_LIPID
REGULATION_OF_GENE_EXPRESSION_IN_ENDOCRINE_COMMITTED_NEUROG3_PROGENITOR_CELLS
FREE_FATTY_ACID_RECEPTORS
RELAXIN_RECEPTORS
OREXIN_AND_NEUROPEPTIDES_FF_AND_QRFP_BIND_TO_THEIR_RESPECTIVE_RECEPTORS
DOPAMINE_RECEPTORS
HORMONE_LIGAND_BINDING_RECEPTORS
CONJUGATION_OF_BENZOATE_WITH_GLYCINE
0 2 4 6 8
t value of GSVA score

Figure S13

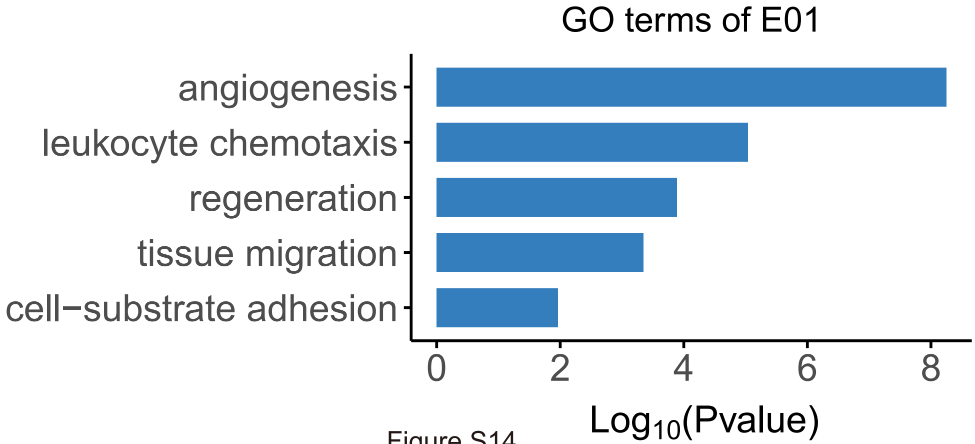
GO terms of E01
angiogenesis
leukocyte chemotaxis
regeneration
tissue migration
cell−substrate adhesion
0
2
4
6
8
Log10(Pvalue)

Figure S14

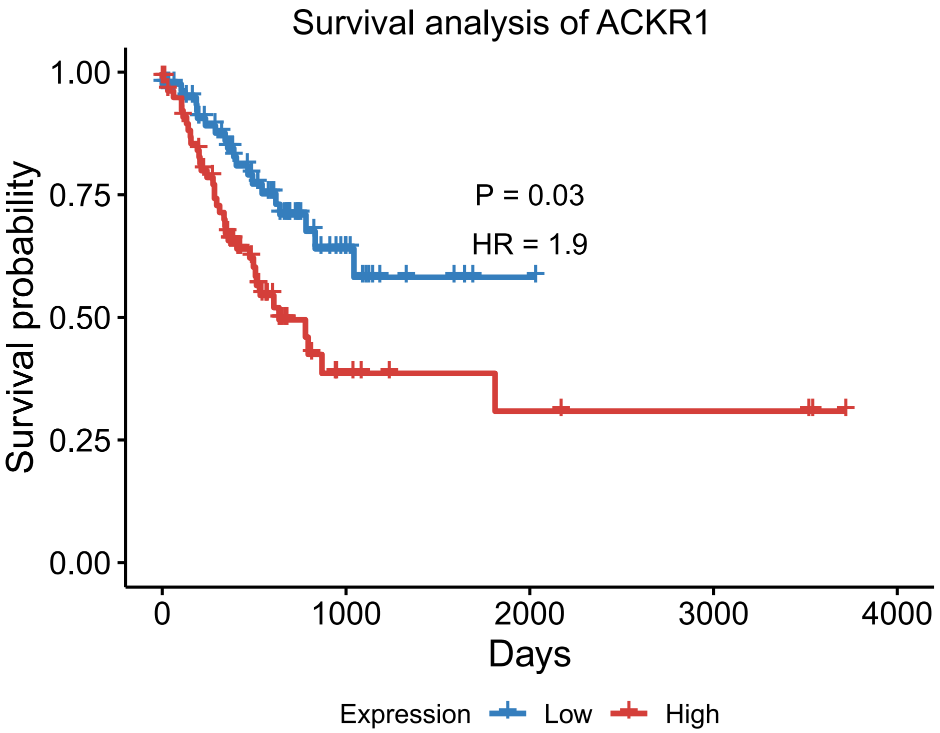

Survival analysis of ACKR1
Survival probability
1.00
0.75
0.50
0.25
0.00
P = 0.03
HR = 1.9
0
1000
2000
3000
4000
Days
Expression
Low
High

Figure S15

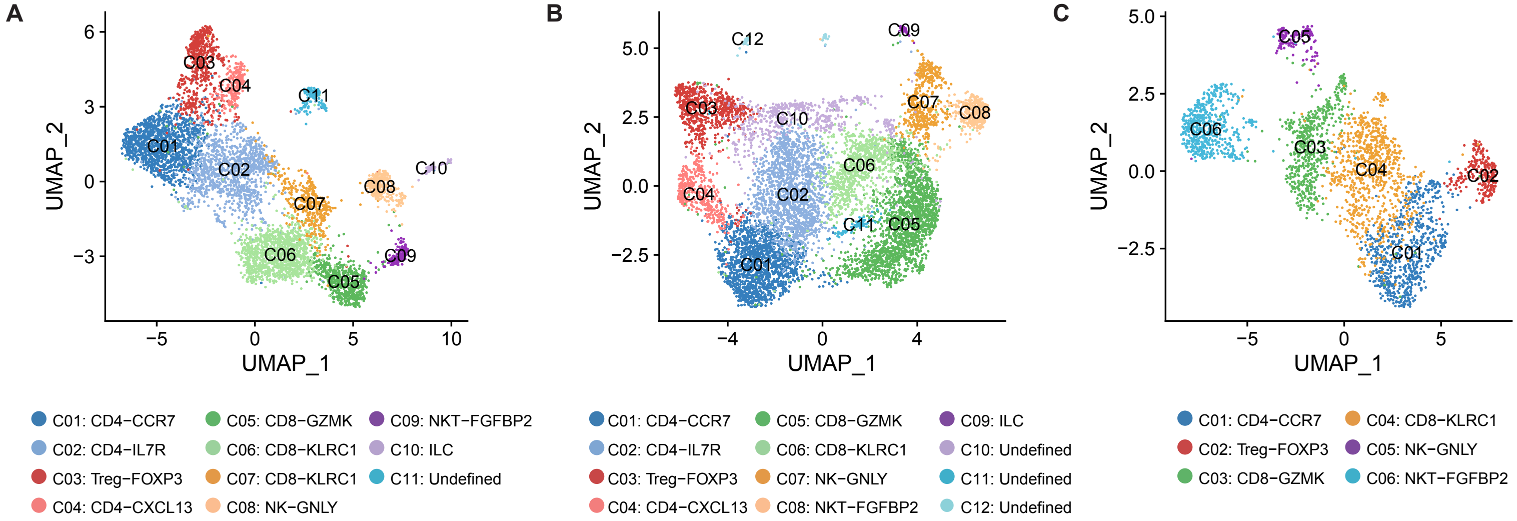
A
B
C
UMAP_1
UMAP_2
C01: CD4−CCR7
C02: CD4−IL7R
C03: Treg−FOXP3
C04: CD4−CXCL13
C05: CD8−GZMK
C06: CD8−KLRC1
C07: CD8−KLRC1
C08: NK−GNLY
C09: NKT−FGFBP2
C10: ILC
C11: Undefined
C01: CD4−CCR7
C02: CD4−IL7R
C03: Treg−FOXP3
C04: CD4−CXCL13
C05: CD8−GZMK
C06: CD8−KLRC1
C07: NK−GNLY
C08: NKT−FGFBP2
C09: ILC
C10: Undefined
C11: Undefined
C12: Undefined
C01: CD4−CCR7
C02: Treg−FOXP3
C03: CD8−GZMK
C04: CD8−KLRC1
C05: NK−GNLY
C06: NKT−FGFBP2

Figure S16

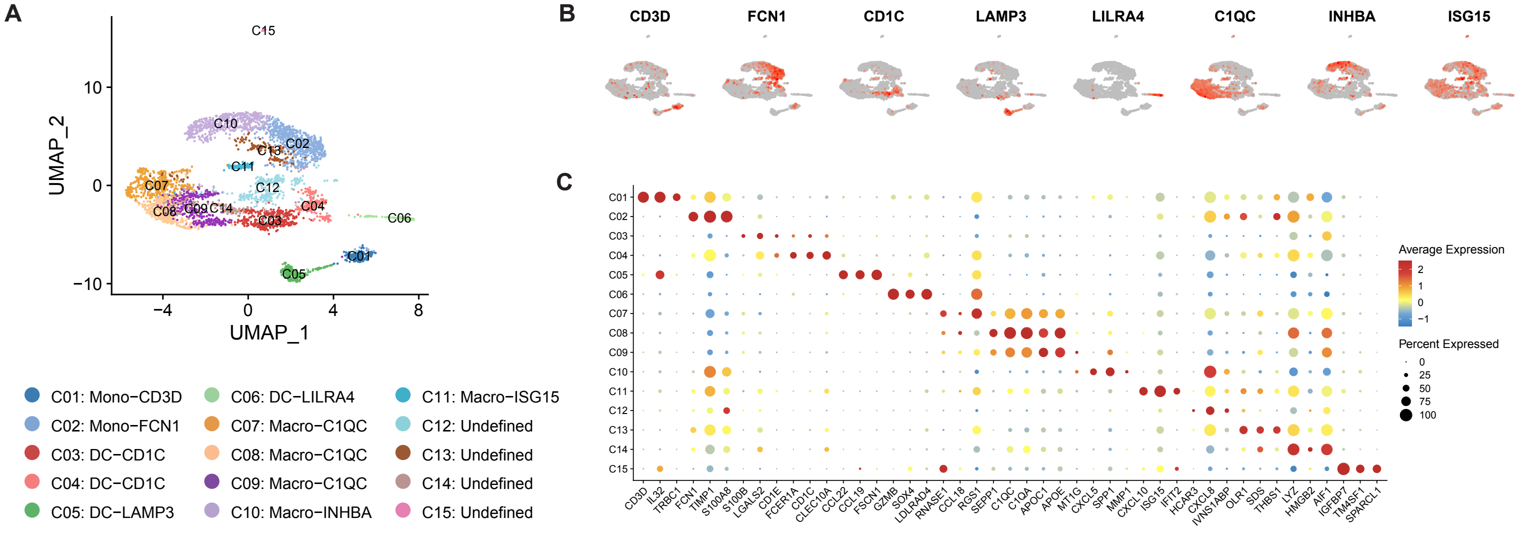
A
B
C
UMAP_1
UMAP_2
C01: Mono−CD3D
C02: Mono−FCN1
C03: DC−CD1C
C04: DC−CD1C
C05: DC−LAMP3
C06: DC−LILRA4
C07: Macro−C1QC
C08: Macro−C1QC
C09: Macro−C1QC
C10: Macro−INHBA
C11: Macro−ISG15
C12: Undefined
C13: Undefined
C14: Undefined
C15: Undefined
CD3D
FCN1
CD1C
LAMP3
LILRA4
C1QC
INHBA
ISG15
Average Expression
Percent Expressed
CD3D IL32 TRBC1 FCN1 TIMP1 S100A8 S100B LGALS2 CD1E FCER1A CD1C CLEC10A CCL22 CCL19 FSCN1 GZMB SOX4 LDLRAD4 RNASE1 CCL18 RGS1 SEPP1 C1QC C1QA APOC1 APOE MT1G CXCL5 SPP1 MMP1 CXCL10 ISG15 IFIT2 HCAR3 CXCL8 IVNS1ABP OLR1 SDS THBS1 LYZ HMGB2 AIF1 IGFBP7 TM4SF1 SPARCL1

Figure S17

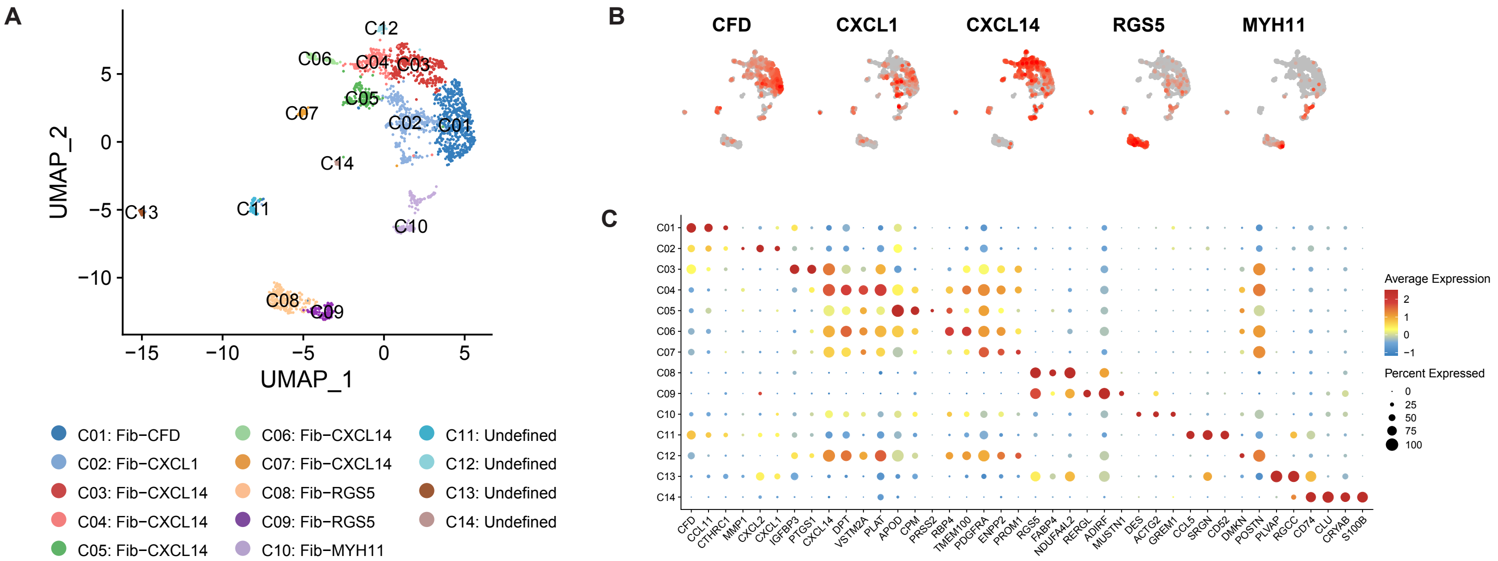
A
B
C
UMAP_1
UMAP_2
C01: Fib−CFD
C02: Fib−CXCL1
C03: Fib−CXCL14
C04: Fib−CXCL14
C05: Fib−CXCL14
C06: Fib−CXCL14
C07: Fib−CXCL14
C08: Fib−RGS5
C09: Fib−RGS5
C10: Fib−MYH11
C11: Undefined
C12: Undefined
C13: Undefined
C14: Undefined
CFD
CXCL1
CXCL14
RGS5
MYH11
Average Expression
Percent Expressed

Figure S18

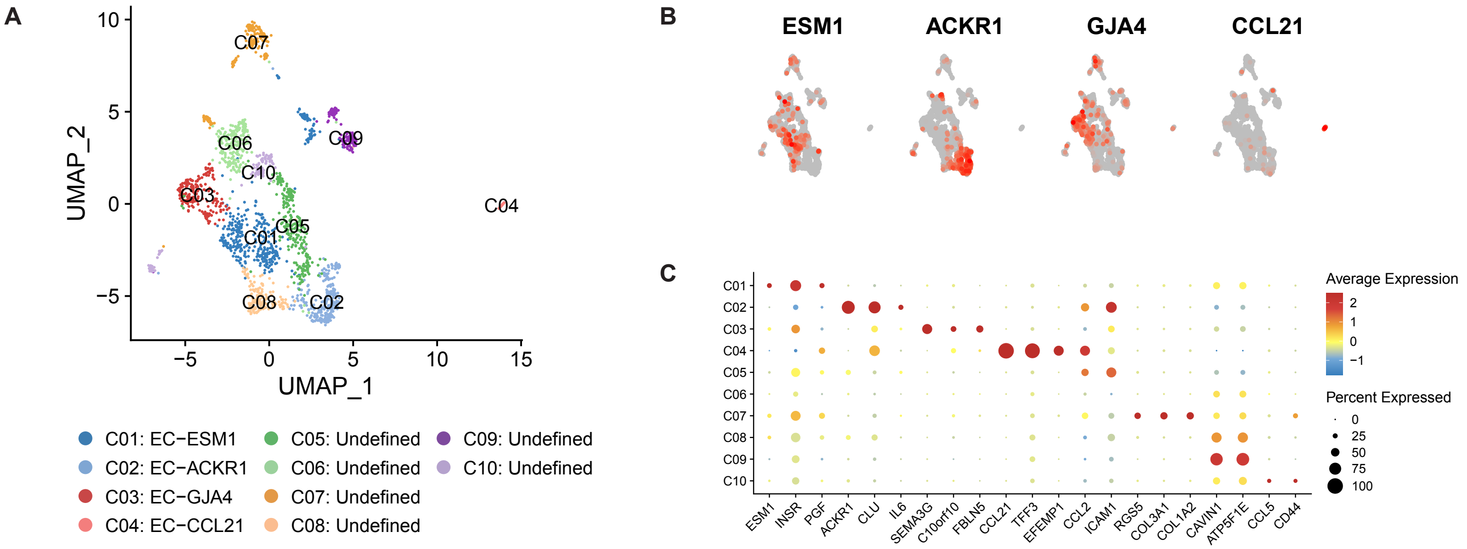
A
UMAP_2
UMAP_1
C01 C02 C03 C04 C05 C06 C07 C08 C09 C10
C01: EC−ESM1
C02: EC−ACKR1
C03: EC−GJA4
C04: EC−CCL21
C05: Undefined
C06: Undefined
C07: Undefined
C08: Undefined
C09: Undefined
C10: Undefined
B
ESM1
ACKR1
GJA4
CCL21
C
Average Expression
Percent Expressed
ESM1 INSR PGF ACKR1 CLU IL6 SEMA3G C10orf10 FBLN5 CCL21 TFF3 EFEMP1 CCL2 ICAM1 RGS5 COL3A1 COL1A2 CAVIN1 ATP5F1E CCL5 CD44

Figure S19

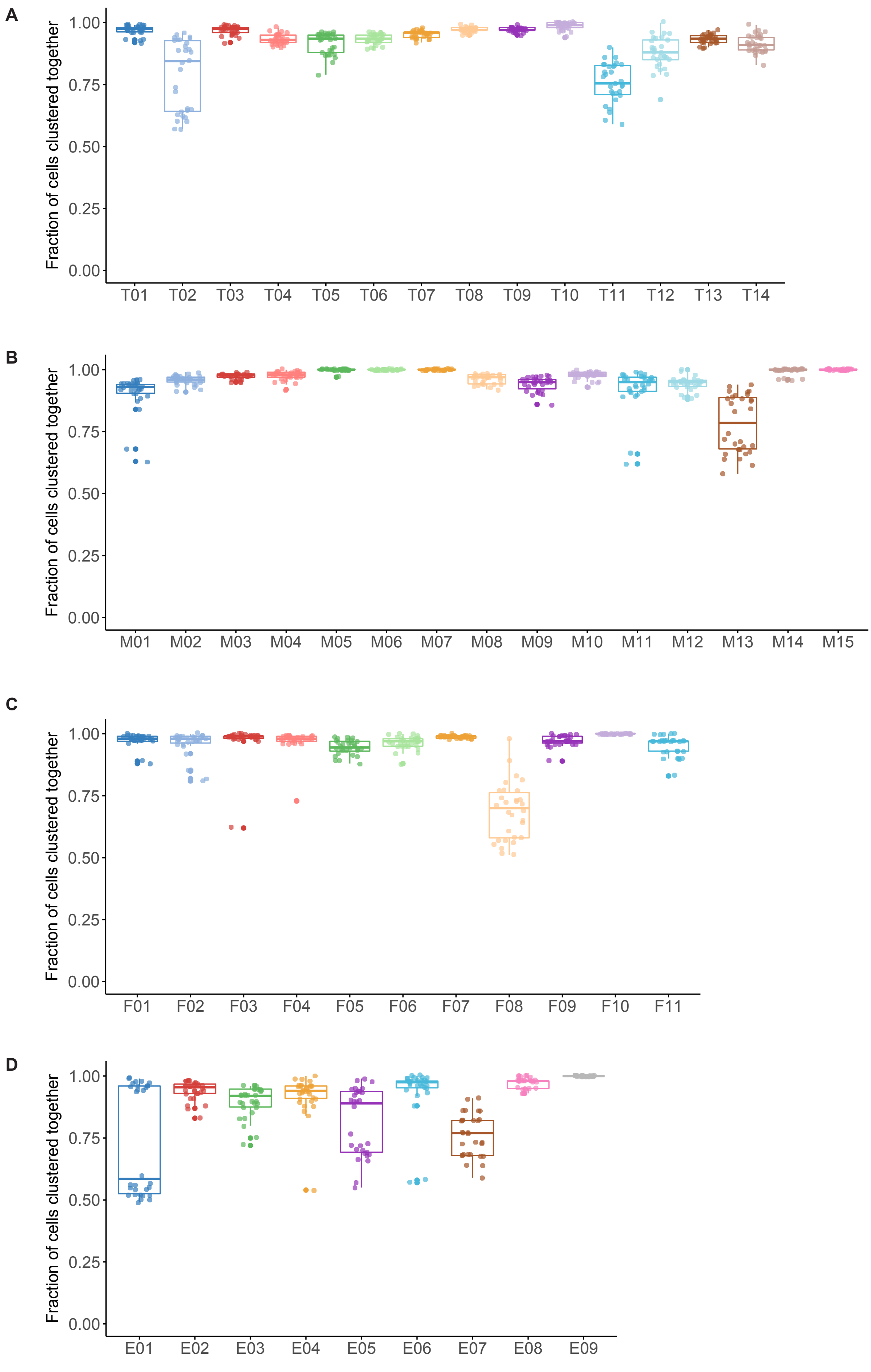

A
B
C
D
Fraction of cells clustered together
1.00
0.75
0.50
0.25
0.00
T01 T02 T03 T04 T05 T06 T07 T08 T09 T10 T11 T12 T13 T14
M01 M02 M03 M04 M05 M06 M07 M08 M09 M10 M11 M12 M13 M14 M15
F01 F02 F03 F04 F05 F06 F07 F08 F09 F10 F11
E01 E02 E03 E04 E05 E06 E07 E08 E09

Figure S20
