# Supplementary material for: Single-cell landscape reveals active cell subtypes and their interaction in the tumor microenvironment of gastric cancer
Source: Theranostics. 2022 May 9;12(8):3818–33. doi: 10.7150/thno.71833 (PMC9131288; doi:10.7150/thno.71833)
Supplement: Supplementary file 2 — Supplementary tables. [file thnov12p3818s2.pdf]

Table S1. The clinical characteristics of nine gastric cancer patients analyzed in this study.

**Patients**	**Sex**	**age**	**Surgery time**	**Tumor location**	**Grade**	**Borrmann classification**	**Lauren classifcation**
D01	male	67	2017/10/12	distal	Poor differeciated	Borrmann III	Diffuse
D02	male	67	2018/1/22	distal	Poor differeciated (Included signet ring cell)	Borrmann III	Diffuse
D03	male	64	2018/4/9	distal	Poor differeciated	Borrmann II	Mixed
P01	male	58	2018/6/21	proximal	Moderately differeciated	Borrmann II	Intestinal
P02	male	72	2017/10/9	proximal	Poor differeciated	Borrmann III	Diffuse
P03	femal e	73	2018/7/19	proximal	Moderately-poor differeciated	Borrmann IV	Mixed
P04	male	68	2018/4/9	proximal	Poor differeciated	Borrmann III	Mixed
P05	male	54	2018/7/26	proximal	Poor differeciated	Borrmann III	Diffuse
P06	male	57	2017/10/16	proximal	Signet ring cell	Borrmann III	Diffuse

*Tumor samples and adjacent normal samples were collected from these nine patients.

Table S2. Top 20 DEGs of T and NK clusters.

Cluster	Label	Genes
T01	CD4-CCR7	CCR7
T02	CD4-IL7R	CCL20,IL7R,KLRB1
T03	Treg-FOXP3	TNFRSF4,IL2RA,BATF,AC133644.2,CARD16,TNFRSF18,MIR4435-2HG,TBC1D4,FOXP3,LAIR2,SOX4,TIGIT,RTKN2,AC017002.1,CTLA4,UGP2,ICA1,ARID5B,LAYN,LINC00152
T04	CD4-CXCL13	CXCL13,ICA1,NR3C1,NMB,METTL8,TOX2,CHN1,FKBP5,MAGEH1,SESN3,TSHZ2,ITM2A,PLIN2
T05	CD8-GZMK	GZMK,CCL4L2,CCL4,CMC1,CST7,GZMH,CRTAM,CCL5,TUBA4A,HLA-DRB1,NKG7,HLA-DPB1,IFNG,SH2D1A,DUSP2
T06	CD8-KLRC1	GZMB,KLRC1,CCL5,CD8A,CD8B,AC092580.4,CCL4,GZMA,ITGA1,GZMH,CCL4L2
T07	Undefined	0
T08	NK-GNLY	GNLY,TYROBP,FCER1G,TRDC,XCL1,GZMA,XCL2,KLRD1,B3GNT7,AREG,KRT86,KLRF1,HOPX,CMC1,KIR2DL4,CTSW,CD7,KLRC2,KLRC1,IFITM2
T09	NKT-FGFBP2	FGFBP2,GNLY,KLRF1,NKG7,PRF1,FCGR3A,KLRD1,GZMH,GZMB,TYROBP,CMC1,CCL3,S1PR5,CTSW,SPON2,CST7,CCL4,PLEK,PLAC8,C1orf21
T10	ILC	LST1,CSF2,IL4I1,HSPB1,AREG,CD83,KRT86,PRMT9,KRT81,KIT,NINJ1,CTNNB1,SPINK2,NCOA7,ZFP36L1,SOX4,XCL1,NFKBIA,TNFRSF18,DLL1
T11	CD8-ISG15	ISG15,IFI6,MX1,IFIT3,IFI44L,STAT1,OASL,LY6E,HERC5,MX2,SAMD9L,RSAD2,OAS1,IFIT1,MT2A,EIF2AK2,XAF1,IFI35,GBP1,ISG20
T12	Proliferating	STMN1,KIAA0101,TUBA1B,TUBB,HIST1H4C,HMGN2,HMGB2,TK1,TYMS,DUT,UBE2C,BIRC5,GNLY,H2AFZ,CENPF,CALM3,RRM2,CKS1B,MKI67,COTL1
T13	Undefined	ADIRF,ANPEP,C19orf33,PIGR,CDHR5,SMIM24,TFF3,IFI27,FCGBP,ELF3,ALDOB,RBP2,MT1G,CLDN4,KRT18,MDK,MUC1,TMC5,KRT19,KRT8
T14	Undefined	IL1RN,RP11-1143G9.4,HLA-DRA,PLAUR,CCL3,PTGS2,FCER1G,CD14,GLUL,VCAN,IER3,MT2A,FTL,NAMPT,APOE,TNFAIP6,FTH1,SAT1,LGALS1,EREG

Table S3. Signature genes of Tregs.

Gene	P value	logFC	pct.1	pct.2	P_adj	Sp_cluster	Sp_celltype	Sp_combined
IL2RA	0	1.552158042	0.334	0.031	0	0.93649799	0.63646789	0.772043327
BATF	0	1.5457564	0.494	0.087	0	0.885751965	0.578597982	0.715887072
TBC1D4	2.73E-273	1.094837302	0.292	0.036	5.94E-269	0.893273933	0.563467492	0.709458119
FOXP3	0	1.058245614	0.24	0.011	0	0.970964041	0.741714286	0.84863296
LAIR2	4.88E-229	1.034221049	0.227	0.023	1.06E-224	0.907661971	0.721448468	0.809216497
TIGIT	9.76E-183	0.957232157	0.38	0.117	2.12E-178	0.719727533	0.814827829	0.765802862
RTKN2	7.27E-209	0.944257767	0.181	0.012	1.58E-204	0.935783426	0.699872286	0.809276767
AC017002.1	1.66E-214	0.940680816	0.156	0.007	3.61E-210	0.97474008	0.552529183	0.73387488
CTLA4	1.41E-199	0.933197506	0.256	0.042	3.07E-195	0.846142212	0.740967962	0.791810754
LAYN	1.18E-187	0.847638888	0.213	0.027	2.56E-183	0.888930818	0.592319055	0.725624326
ICOS	2.04E-152	0.842742464	0.408	0.153	4.44E-148	0.661455884	0.827837759	0.739985241
TNFRSF9	2.92E-155	0.831535919	0.208	0.034	6.37E-151	0.842143412	0.624113475	0.724977966
AC002331.1	1.20E-140	0.8082186	0.196	0.033	2.60E-136	0.83421431	0.823586745	0.828883495
CXCR6	6.47E-120	0.761510183	0.303	0.1	1.41E-115	0.684498566	0.850923483	0.763187987

*logFC is log fold change. P value and logFC were produced in differential expression analysis. P_adj is adjusted P value. pct.1 is the percent of expressed cells in Tregs. pct.2 is the percent of expressed cells in other T and NK clusters.

Table S4. Top 20 DEGs of myeloid clusters.

Cluster	Label	Genes
M01	Mono-CD14	C1QC,C1QB,C1QA,IL23A,SDS,FCGBP
M02	Mono-CD16	IFITM2,FCGR3B,HCAR3,ADM,S100A8,CSF3R,RP11-670E13.6,MNDA,IFITM1,ANP32A,FPR2,S100A9,IFITM3,IVNS1ABP,CD69,DUSP1,NAMPT,SELL,GCA,FPR1
M03	Mono-CD3D	IL32,KLRB1,CD3D,CCL5,ETS1,TRAC,CD2,GZMA,LTB,CD7,TRBC2,TRBC1,CD3E,RORA,CD3G,ZFP36L2,ITM2A,GNLY,CNOT6L
M04	DC-CD1C	FCER1A,HLA-DQA1,HLA-DQB1,CD1C,HLA-DPB1,CLEC10A,PPA1,HLA-DPA1,HLA-DQA2,CST7,CD1E,JAML,HLA-DRA,PKIB,CD74,HLA-DRB1,PLAC8,GPR183,AXL,HLA-DQB2
M05	DC-CLEC9A	DNASE1L3,CPVL,SNX3,CLEC9A,LGALS2,CPNE3,TACSTD2,C1orf54,HLA-DPA1,HLA-DPB1,IDO1,S100B,CST3,HLA-DQA1,NET1,HLA-DQA2,LSP1,GSTP1,HLA-DQB1,RAB11FIP1
M06	DC-LAMP3	CCL22,CCR7,CCL19,TXN,FSCN1,BIRC3,EBI3,CRIP1,IDO1,LAMP3,CCL17,RAMP1,TBC1D4,ID2,DUSP5,LSP1,CST7,GRSF1,RAB9A,NUB1
M07	DC-LILRA4	GZMB,PTGDS,SOX4,C12orf75,LDLRAD4,TSPAN13,IRF4,NR3C1,CLIC3,ITM2C,PPP1R14B,IRF7,TCF4,PTPRS,SEL1L3,PLD4,CXCR4,GPR183,LILRA4,ZC3HAV1
M08	Macro-INHBA	CXCL5,IL6,CXCL1,CXCL3,CCL3L3,CCL20,CCL3,PTGS2,INHBA,IL1RN,MMP1,CXCL8,TNIP3,CXCL2,IL1A,IL1B,C15orf48,SDC2,SPP1,TIMP1
M09	Macro-C1QC	C1QC,C1QB,C1QA,IL23A,SDS,FCGBP
M10	Macro-OTOA	APOE,APOC1,SEPP1,GPNMB,RNASE1,C1QB,C1QA,CTSD,C1QC,CD9,LIPA,LGMN,TREM2,PLD3,MSR1,MT1G,ACP5,SPP1,CTSB,FABP5
M11	Macro-ISG15	CXCL10,ISG15,GBP1,CXCL11,CXCL9,IDO1,FAM26F,IFIT2,APOBEC3A,ISG20,GBP4,IFI27,STAT1,IFIT3,VAMP5,LAP3,RSAD2,IFI6,MX1,TNFSF10
M12	Undefined	HSPA1B,HSPA6,DNAJB1,HSPA1A,HSPH1,ZFAND2A,HSPB1,HSP90AA1,BAG3,HSPE1,HILPDA,JUN,HSPD1,UBB,CCL4,HES1,CCL3,PLIN2,FOS,DNAJA1
M13	Undefined	APOA1,RP11-1143G9.4,C1orf56
M14	Proliferating	TUBA1B,RETN,HIST1H4C,STMN1,TUBB,TOP2A,HMGB2,UBE2C,MKI67,CENPF,CCDC109B,KPNA2,VCAN,NUSAP1,HMGN2,HMGB1,CKS1B,RAN,CCT5
M15	Undefined	IGFBP7,RGS5,SPARCL1,CALD1,BGN,TAGLN,MGP,SPARC,MYL9,ACTA2,COL1A2,ASPN,COL3A1,COL4A1,DCN,FN1,COL6A2,IGFBP5,COL1A1,CTGF

Table S5. Signature genes of DC-LAMP3.

Gene	P value	logFC	pct.1	pct.2	P_adj	Sp_cluster	Sp_celltype	Sp_combined
CCL22	2.19E-123	3.015127592	0.778	0.029	4.79E-119	0.99458972	0.521091811	0.719911494
IDO1	3.08E-73	2.041958535	0.741	0.061	6.72E-69	0.950901713	0.517699115	0.701627376
CCL17	6.07E-31	1.852755796	0.315	0.017	1.33E-26	0.964941389	0.672839506	0.805760937
CSF2RA	7.74E-45	1.313163791	0.769	0.242	1.69E-40	0.871200442	0.876360809	0.873776816
TVP23A	1.86E-40	0.892047635	0.333	0.009	4.06E-36	0.981064022	0.616	0.777390145

*logFC is log fold change. P value and logFC were produced in differential expression analysis. P_adj is adjusted P value. pct.1 is the percent of expressed cells in DC-LAMP3. pct.2 is the percent of expressed cells in other myeloid clusters.

Table S6. Top 20 DEGs of fibroblast clusters.

Cluster	Label	Genes
F01	Fib-RGS5	RGS5,NDUFA4L2,COL4A1,COL4A2,PRSS23,CD36,COX4I2,HIGD1B,LINC00152,CHN1,NOTCH3,MIR4435-2HG,IGFBP2,EGFL6,GJA4,ITGA1,ANGPT2,ACTA2,ARHGDIB,CRIP1
F02	Fib-MYH11	ADIRF,MYH11,CRIP1,ACTA2,TAGLN,PLN,MYL9,TINAGL1,RERGL,IL6,SORBS2,MT1M,MAP3K7CL,MT1A,ACTG2,DSTN,BCAM,CSRP2,SNCG,TPM2
F03	Fib-CXCL14	CXCL14,POSTN,F3,PLAT,PDGFRA,BMP4,NSG1,TMEM176B,ENHO,PDGFD,TRPA1,FENDRR,AGT,C8orf4,TMEM176A,HSD17B2,CCL11,PLPP3,RGS10,EMID1
F04	Fib-CTHRC1	SFRP4,CTHRC1,GREM1,COL8A1,ITGBL1,SULF1,INHBA,BGN,TIMP3,COL1A1,TIMP1,COL3A1,THBS2,HOPX,COL10A1,SERPINE2,LOXL1,COL1A2,FAP,PPIC
F05	Fib-C7	C7,APOD,IGF1,DPT,RARRES1,FBLN1,PTGDS,C1QTNF3,NPPC,CXCL12,KCNN3,CYP1B1,ADH1B,OGN,THBS4,MGP,DNAJB1,SCN7A,JUN,KLF4
F06	Fib-CFD	PLA2G2A,CFD,CLU,IGFBP6,SFRP2,SLPI,PI16,SFRP1,MFAP5,TNXB,GSN,CCDC80,ITM2A,FBLN1,GPNMB,DCN,MGST1,FBLN2,PLAC9,RARRES1
F07	Resting	C1orf56,CTNNB1,HSPG2
F08	Fib-COL7A1	MMP1,CXCL1,MMP3,CXCL5,CXCL6,CXCL8,IL24,AREG,MT2A,G0S2,CHI3L1,FTH1,CXCL3,PLAU,STC1,C15orf48,TNFAIP6,SAA1,HLA-DRA,MT1E
F09	Undefined	GZMA,CCL5,GNLY,RGS1,CD7,CD52,SRGN,CXCR4,KLRB1,PTPRC,CD69,TRAC,LTB,CD3D,TRBC2,CD3E,CREM,CD2,TNFRSF4,CORO1A
F10	Undefined	CLDN5,ACKR1,PLVAP,RAMP2,SELE,RNASE1,AQP1,EGFL7,PCAT19,VWF,FABP5,CLEC14A,TM4SF1,CSF3,CD74,HLA-DRB1,EMCN,RAMP3,IL6,FKBP1A
F11	Proliferating	HIST1H4C,IGFBP5,PTTG1,LTBP4,UBE2C,CXCL14,TOP2A,CENPF,ABCA8,TAF3,HSPA1B,HMGN2,KIN,ADAM28,STMN1,H2AFZ,VEGFB,ANKRD13A,EMILIN1,BMP4

Table S7. Signature genes of Fib-CTHRC1.

gene	p_val	avg_logFC	pct.1	pct.2	p_val_adj	Sp_cluster_score	Sp_celltype_score	Sp_combined_score
SFRP4	1.03E-181	1.964230689	0.662	0.103	2.24E-177	0.897090676	0.871523179	0.88421452
GREM1	8.97E-135	1.369409973	0.565	0.098	1.95E-130	0.879436776	0.864035088	0.871701917
COL8A1	3.58E-178	1.34544356	0.724	0.166	7.80E-174	0.878475741	0.915254237	0.896676444
ITGBL1	8.91E-124	1.078041206	0.55	0.093	1.94E-119	0.851264334	0.877358491	0.864212932
SULF1	3.94E-87	1.01097902	0.575	0.188	8.58E-83	0.75483064	0.812202097	0.782991079
THBS2	9.98E-89	0.906736262	0.556	0.159	2.17E-84	0.776309808	0.817887931	0.796827725
COL10A1	3.17E-116	0.882626113	0.373	0.026	6.91E-112	0.940852664	0.842424242	0.890279222
LOXL1	8.59E-87	0.822587931	0.581	0.181	1.87E-82	0.773649235	0.799432356	0.786435141
FAP	3.11E-108	0.810742685	0.568	0.125	6.77E-104	0.818286239	0.688935282	0.750830381
FGF7	1.15E-57	0.738641293	0.646	0.286	2.50E-53	0.575668416	0.890160183	0.715847122
ASPN	6.08E-65	0.71964385	0.75	0.366	1.32E-60	0.548776814	0.893512852	0.700242198

*logFC is log fold change. P value and logFC were produced in differential expression analysis. P_adj is adjusted P value. pct.1 is the percent of expressed cells in Fib-CTHRC1. pct.2 is the percent of expressed cells in other fibroblast clusters.

Table S8. Top 20 DEGs of endothelial clusters.

Cluster	Label	Genes
E01	EC-ESM1	ESM1,CA2,LINC00152,ANGPT2,SPARC,MIR4435-2HG,COL4A1,IGFBP3,RGCC,FLT1,EDNRB,INSR,RBP7,KDR,RGS3,HTRA1,LBH,COL4A2,GABRD,THY1
E02	EC-ACKR1	IL6,VCAM1,SELE,ACKR1,CCL2,CXCL2,SOD2,CLU,CXCL3,HLA-DRB1,HLA-DRA,CEBPD,ADIRF,ICAM1,CCL14,CPE,NPC2,POSTN,NNMT,NCOA7
E03	EC-CTHRC1	SPP1,THBS1,CTHRC1,COTL1,LGALS1,COL6A2,F3,SELM,SERPINE1
E04	EC-CD36	TMEM88,SDPR,FOS,SGK1,MGLL,CD36,CD320,TXNIP
E05	EC-GJA4	SLC9A3R2,SLC6A6,C10orf10,CXCL12,IGFBP5,EMP3,PI16,GJA4,ATP13A3,PODXL,FABP4,HEY1,SRP14,EDN1,S100A4,PPP1R14A,ADAMTS6,IGF2,SAT1,CRIP1
E06	Undefined	HSPA1B,EGFL7
E07	EC-CXCL10	CXCL10,CXCL11,GBP1,ISG15,CXCL9,GBP4,MX1,PARP14,RSAD2,LY6E,LAP3,IFI44L,WARS,IL32,IFI6,IFIT3,BST2,TAP1,TNFSF10,IFIT1
E08	Undefined	CXCR4,RGS1,CD52,S100A4,CD3D,PTPRC,TRAC,CCL5,GZMA,CREM,KLRB1,CD2,CD7,MZB1,GPR183,TRBC2,SPOCK2,CD69,LTB,CD3E
E09	EC-CCL21	CCL21,TFF3,NTS,EFEMP1,CLU,TFPI,AKAP12,FABP4,TIMP1,APOD,BGN,CXCL1,LYVE1,RBP1,ADIRF,CTSD,MMRN1,SNCG,COLEC12,RARRES2

Table S9. Signature genes of EC-ESM1.

gene	p_val	avg_logFC	pct.1	pct.2	p_val_adj	Sp_cluster_score	Sp_celltype_score	Sp_combined_score
ESM1	4.83E-51	1.926314886	0.357	0.041	1.06E-46	0.943958333	0.650862069	0.783828217
KDR	7.04E-33	0.803062836	0.539	0.18	1.54E-28	0.69577381	0.834782609	0.762115395
GABRD	2.23E-36	0.719797769	0.301	0.034	4.87E-32	0.907130952	0.848684211	0.87742106

*logFC is log fold change. P value and logFC were produced in differential expression analysis. P_adj is adjusted P value. pct.1 is the percent of expressed cells in EC-CTHRC1. pct.2 is the percent of expressed cells in other EC clusters.
